# Trade policy uncertainty and stock price crash risk in China: The moderating role of marketization and digital transformation

**DOI:** 10.1371/journal.pone.0338820

**Published:** 2025-12-26

**Authors:** Chengwei Liu, Tajul Ariffin Masron, Haiyan Huo

**Affiliations:** 1 School of Business, Guangxi City Vocational University, Chongzuo, Guangxi, China; 2 School of Management, Universiti Sains Malaysia, Penang, Malaysia; 3 Azman Hashim International Business School, Universiti Teknologi Malaysia, Kuala Lumpur, Malaysia; 4 School of Business Management, Xingtai University, Xingtai, Hebei, China; OP Jindal Global University, INDIA

## Abstract

This paper investigates the link between trade policy uncertainty (TPU) and stock price crash risk in Chinese listed firms. Using a novel firm-level TPU index derived from annual reports between 2001 and 2023, we show that heightened TPU significantly elevates crash risk, robust across multiple specifications and measures. The effect is particularly pronounced in private firms, those with CEO duality, internationalized firms, and those audited by non-Big Four auditors. Mechanism tests reveal that TPU exacerbates crash risk through discretionary accruals, suppressed exports, investor sentiment distortions, analyst forecast bias, and information asymmetry. Importantly, market liberalization and digital transformation act as effective buffers. Our findings highlight TPU as a key determinant of firm-level fragility and extend the literature on uncertainty by uncovering the micro-level channels through which trade policy shocks destabilize capital markets, offering actionable insights for policymakers and investors.

## 1. Introduction

**Fig 1:**
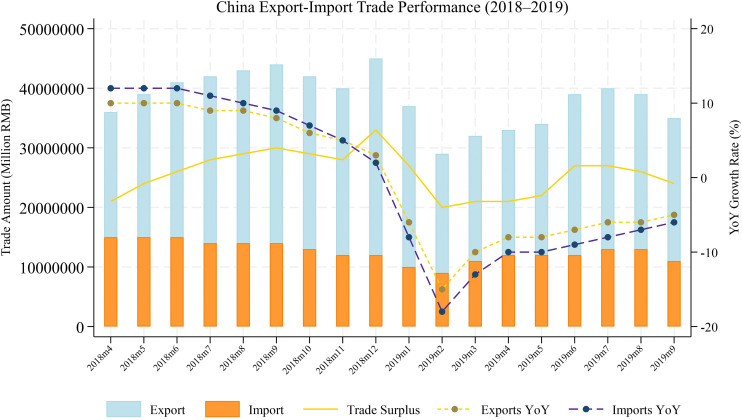
Trade dynamics between China and the U.S. after 2018 trade war.

**Fig 2:**
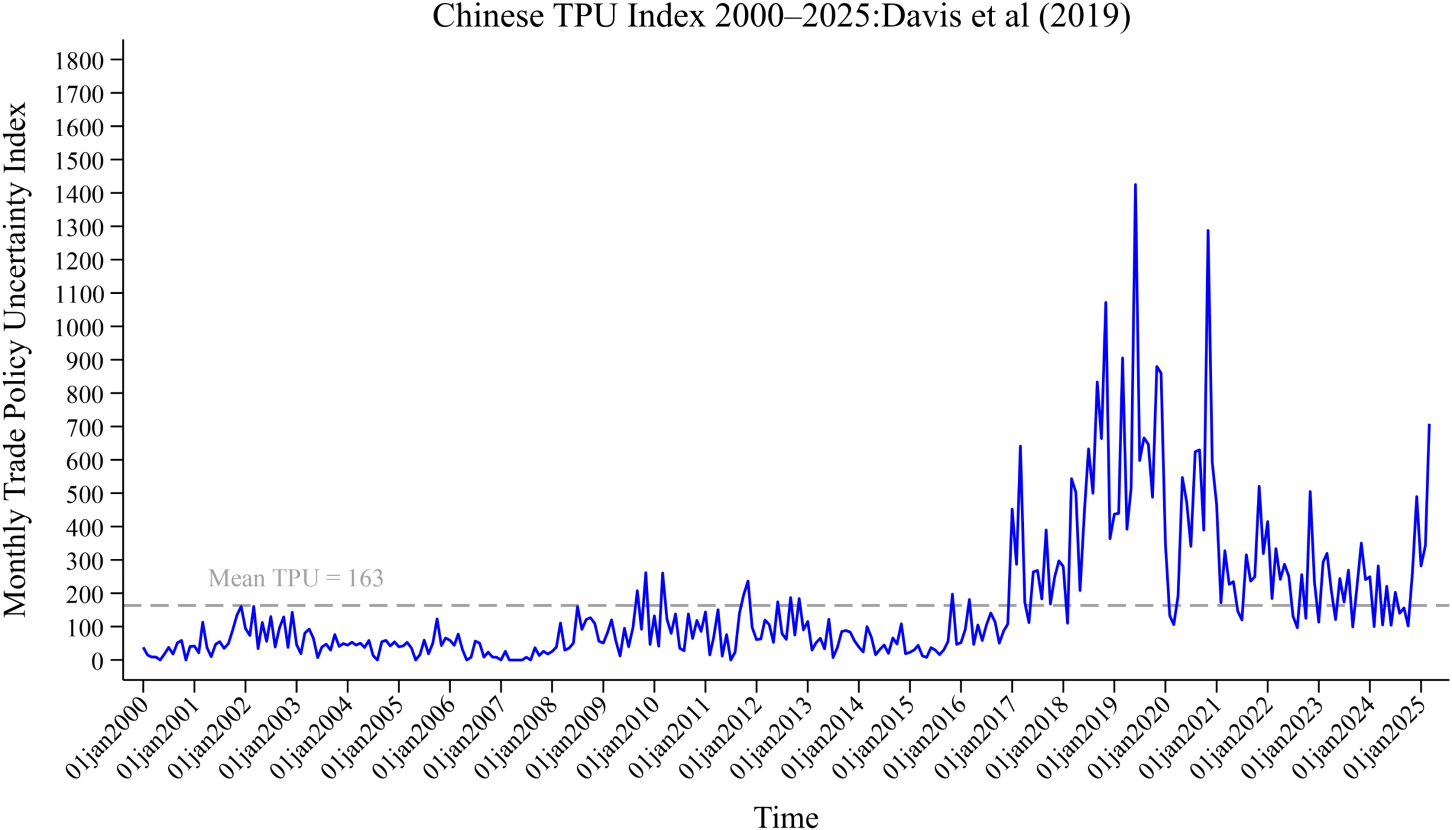
Monthly trade policy uncertainty index for China by Davis et al. [2].

The outbreak of the U.S.–China trade war in 2018 profoundly and permanently reshaped the trade structure between the two countries. As shown in Fig 1, both the level and year-on-year growth rate of China’s exports declined markedly in the first half of 2019, with export growth turning negative between June and August; imports experienced even sharper contractions. Since then, China’s trade policy uncertainty (TPU) has exhibited a persistent upward trend [1]. As illustrated in Fig 2, the TPU index [2] has repeatedly exceeded its historical mean, reflecting heightened anxieties among firms and investors regarding future policy direction. In April 2025, a new and intensified round of tariff conflict erupted between China and the United States. The U.S. government announced a 25% additional tariff on a broad range of core Chinese exports. On April 7, the first trading day following the announcement, Chinese A-share markets experienced a sharp selloff: the Shanghai Composite plunged 7.34%, the Shenzhen Component Index fell by 9.66%, and the ChiNext Index tumbled 12.50%—its steepest single-day drop in a decade. This episode underscores TPU’s role as a major external shock capable of triggering nonlinear and disorderly adjustments in capital markets [3, 4], inflicting substantial losses on investors [5].

Due to its profound implications for capital markets, trade policy uncertainty has attracted widespread scholarly attention. Prior studies document that elevated TPU significantly depresses stock returns [4], deteriorates market liquidity [6], amplifies volatility [7, 8], strengthens price comovement [9], and intensifies herd behavior [10]. Under heightened TPU, corporate managers, aiming to prevent adverse market reactions, often opt to withhold or delay the disclosure of unfavorable information [11, 12]. However, such concealment practices can lead to the accumulation of negative signals, forming a “bad news damming effect” [13, 14]. When these suppressed signals are eventually released, they tend to trigger sharp market corrections and elevate the risk of stock price crashes [11, 15]. In parallel, TPU may also undermine price efficiency by reducing market liquidity [6, 16] and exacerbating investor sentiment biases and irrational trading behavior [17, 18]. These dynamics collectively impair the market’s ability to incorporate firm-specific fundamentals, thereby disrupting information transmission mechanisms and increasing the likelihood of nonlinear crash events [19, 20].

However, a central unanswered question in the crash-risk literature is whether trade policy uncertainty (TPU) independently amplifies stock price crash risk, and if so, via which firm-level transmission channels. Unlike broad economic policy uncertainty (EPU), TPU originates specifically from fluctuations in tariffs, trade agreements, and non-tariff barriers and directly modifies firms’ export access, cost structure, and supply chain stability [1, 21]. These characteristics make TPU especially relevant to information opacity and negative news accumulation—key ingredients in crash formation—beyond what EPU can explain. To address this, we test the independent effect of TPU on crash risk and explore mechanisms such as discretionary accruals, export, analyst forecast bias, and information asymmetry. Empirically, we exploit firm-level exposure to trade policy shocks to identify heterogeneous responses and trace how TPU translates into crash risk through these channels.

Assuming that TPU significantly elevates stock price crash risk, it becomes essential to explore moderating mechanisms that may cushion this effect. Prior studies identify two critical buffers: marketization [22] and digital transformation [23]. At the institutional level, deeper marketization reforms enhance transparency and legal safeguards, foster standardized disclosure, strengthen the interpretative role of intermediaries, and facilitate more efficient capital allocation, thereby anchoring expectations, mitigating sentiment-driven overreactions, and reinforcing market liquidity and resilience [22, 24]. At the firm level, digital transformation improves the timeliness and completeness of disclosure, reduces information acquisition costs, and strengthens organizational flexibility, while intelligent risk-monitoring systems enhance ex-ante recognition and absorptive capacity. Moreover, digital infrastructure optimizes liquidity distribution and market microstructure, providing more robust shock-absorption mechanisms and reducing systemic vulnerabilities under TPU shocks [23, 25].

This study demonstrates that trade policy uncertainty (TPU) significantly increases the risk of stock price crashes among Chinese listed firms, and the effect remains robust across a range of alternative specifications and tests. The heterogeneity analysis shows that private firms, those with CEO duality, internationalized enterprises, and firms audited by non-Big Four auditors are particularly vulnerable to TPU shocks, while higher levels of marketization and digital transformation mitigate these adverse effects. Mechanism tests further reveal that TPU amplifies crash risk primarily by encouraging discretionary accruals, export, investor sentiment, analyst forecast bias, and information asymmetry. Taken together, the findings highlight that TPU is not merely a macro-level policy disturbance but a critical determinant of firm-level financial fragility. By uncovering these channels, the paper contributes new micro-level evidence on how trade-related policy uncertainty undermines capital market stability and offers insights for policymakers seeking to reduce systemic risks associated with volatile trade environments.

This paper makes three contributions to the literature. First, it provides the first systematic evidence that trade policy uncertainty significantly increases the risk of stock price crashes, and it further identifies the mechanisms—ranging from discretionary accruals and export to investor sentiment, analyst forecast bias, and information asymmetry—through which this effect materializes, and further uncovers the heterogeneous firm responses. Second, it develops a novel firm-level measure of TPU by applying textual analysis to corporate disclosures, thereby capturing micro-level exposure that is overlooked by macro-based indices and enabling a more precise identification of risk transmission. Third, it examines the moderating roles of external institutional conditions and internal adaptive capabilities, showing that higher levels of regional marketization and firms’ digital transformation serve as important buffers that attenuate the destabilizing consequences of TPU. Collectively, these contributions enrich our understanding of how trade-related uncertainty permeates financial markets and offer policy-relevant insights into strengthening market resilience.

The remainder of the paper is organized as follows. Section 2 summarizes related literature on the relationship between trade policy uncertainty and stock price crash risk, and two mitigation strategies. Section 3 describes the data, variable constructions, and estimating methods. Section 4 presents the main results. The additional tests are reported in Section 5, and Section 6 concludes the study.

## 2. Literature review and hypothesis development

### 2.1. Trade policy uncertainty and its economic effect

Trade policy plays a pivotal role in shaping uncertainty in China’s external economic trajectory [26]. It functions both as a stabilizer that reduces environmental ambiguity and as a shock amplifier that heightens market volatility [27]. Crucially, its effect is not determined by whether a policy is favorable or unfavorable in substance, but by whether it embodies clear, stable, and predictable rules [21]. The core source of trade policy uncertainty lies in the absence of rule continuity, transparency, and coherence [28]. When policies lack credible forward guidance, exhibit frequent reversals, or reflect geopolitical frictions, uncertainty escalates sharply [27]. For instance, during the U.S.–China trade war, the erratic imposition and revision of U.S. tariffs—combined with opaque enforcement standards and negotiation breakdown risks—led to heightened uncertainty over firms’ cost structures and institutional expectations [21]. Similarly, the EU’s Carbon Border Adjustment Mechanism (CBAM), introduced amid fragmented regulatory standards, intensified compliance uncertainty for Chinese exporters in carbon-intensive sectors [29]. So trade policy uncertainty (TPU) refers to the risk that prospective changes in tariffs, quotas, or trade agreements may alter the expected conditions upon which international trade and investment decisions are based [21]. As a result, it introduces ex-ante uncertainty into the optimization problems faced by firms and investors [16].

Trade policy uncertainty (TPU) and economic policy uncertainty (EPU) are closely related yet fundamentally distinct. Economic policy uncertainty (EPU) is a broad concept that captures unpredictability in fiscal, monetary, and regulatory environments, and has been shown to affect macroeconomic outcomes such as investment, volatility, and crash risk [7, 30]. Trade policy uncertainty (TPU), by contrast, constitutes a specific dimension of EPU that directly concerns the stability of tariffs, trade agreements, and non-tariff barriers [21]. While TPU is conceptually nested within EPU, it is analytically distinct and non-substitutable [1]. This is because trade policy shocks alter firms’ external market access, cost structures, and supply chain resilience in ways that general macroeconomic policy uncertainty does not [28]. For instance, unexpected tariff escalations or abrupt changes in trade rules can trigger concentrated losses in export-oriented sectors and amplify firm-level crash risk, even after controlling for broader EPU [31]. Therefore, by constructing a firm-level TPU measure, this study highlights a unique mechanism of uncertainty transmission, complementing but not replicating the effects of EPU [1].

At the firm level, the impact of TPU is particularly pronounced, as uncertainty over trade rules directly shapes corporate strategic orientation, investment decisions, and risk exposure. Elevated TPU raises the option value of waiting, leading firms to delay or forego export entry and to increase the likelihood of exit from foreign markets, thereby compressing extensive-margin growth and undermining internationalization strategies [32]. It also reshapes firms’ investment behavior, as uncertainty over trade costs and market access reduces capital expenditures, curtails R&D intensity, and weakens long-term innovation incentives [30, 31]. In addition, TPU heightens financing constraints by amplifying cash flow volatility and increasing the cost of external capital, thereby constraining firms’ capacity to absorb shocks and adapt flexibly to changing environments [33]. Export-oriented enterprises, in particular, are more vulnerable to these dynamics since tariff shocks and renegotiation risks directly erode profitability and destabilize supply chains [21]. Taken together, these underscore that TPU does not merely operate as a macroeconomic disturbance but fundamentally alters firm-level resource allocation, strategic orientation, and resilience, shaping both short-term performance and long-term competitiveness.

### 2.2. Trade policy uncertainty and stock price crash risk

A growing body of literature finds that economic uncertainty significantly increases the risk of stock price crashes [11, 13, 15, 34, 35]. Building on this foundation, this study explores the mechanisms through which trade policy uncertainty (TPU) exacerbates crash risk, focusing on three interrelated dimensions: information asymmetry [17], investor behavioral biases [18], and the deterioration of trading mechanisms [36].

First, TPU heightens firm–investor information asymmetries by disrupting the generation, transmission, and interpretation of information, thereby increasing crash risk [37]. On the one hand, heightened TPU weakens managerial incentives to disclose unfavorable information [38], prompting selective disclosure behavior during periods of rising uncertainty [15]. This leads to a “bad news hoarding” effect, where the accumulation of undisclosed information increases the likelihood of abrupt price corrections upon release [37]. On the other hand, TPU undermines external informational transparency [39, 40], particularly for firms highly exposed to policy regimes, thereby raising the cost of accurate information acquisition for investors and amplifying divergence in beliefs and pricing errors [41]. Furthermore, TPU impairs the market’s ability to incorporate new information efficiently [28, 42], causing fundamental changes to be reflected only after critical thresholds are breached, which results in discontinuous price adjustments [5].

Second, from the perspective of market participants’ behavior, TPU acts as an exogenous shock that amplifies cognitive distortions and behavioral biases [18, 31]. Elevated TPU strengthens managerial overconfidence, particularly in firms with highly centralized power structures, increasing the likelihood of distorted judgment and suppression of bad news [43]. Investors, likewise, exhibit heightened emotional responses under TPU [44], with panic and herding behaviors driving synchronized mass sell-offs in response to negative signals [9, 45]. In addition, analyst forecasts become more error-prone under uncertain policy environments [46], and heightened market attention to firm outlooks can result in forecasts that deviate from fundamentals, misleading investor expectations and intensifying crash susceptibility [14, 47, 48].

Finally, TPU impairs market microstructure and trading mechanisms, thereby distorting price discovery [36]. Specifically, TPU reduces liquidity provision [16] by disrupting quoting behavior and increasing the risk of adverse selection, discouraging limit order submission and reducing market depth [36]. In such illiquid conditions, even minor negative news can trigger sharp price swings, amplifying market reactions [49]. TPU also suppresses investor trading activity [50], inducing precautionary behavior [51], shrinking order flow and weakening transaction intensity [20]. In the absence of adequate trading volume, the efficiency of the price discovery process deteriorates, causing delayed responses to risk signals and the buildup of latent crash risk [6, 20]. Based on the above analysis, we propose the following hypothesis:

Hypothesis 1: All else equal, trade policy uncertainty significantly increases the risk of stock price crashes.

### 2.3. Moderating factors

#### 2.3.1. Improving the marketization.

Marketization, as a key institutional determinant of systemic risk in capital markets, may play a moderating role in the relationship between trade policy uncertainty (TPU) and stock price crash risk [22, 24, 52–54]. This study examines such moderation through three channels: institutional stability, information transparency, and the financial system’s capacity to absorb shocks. First, marketization fosters institutional predictability, thereby stabilizing firm behavior under TPU. Specifically, a more market-oriented policy framework enhances the openness and consistency of policymaking [55], while a sound legal system ensures contract enforcement and buffers external shocks [56]. Meanwhile, reduced administrative intervention mitigates firms’ reliance on non-market signals when adjusting valuations [57]. Second, marketization improves the structure and perception of information disclosure, thereby reducing noise and distortions caused by TPU [58]. In highly marketized environments, disclosure practices are more standardized, limiting the hoarding of bad news [36]; more professional intermediaries interpret policy signals more efficiently, curbing misperceptions [48]; and lower information costs promote cognitive convergence, discouraging sentiment-driven trading [59]. Third, marketization enhances capital allocation efficiency and liquidity depth, strengthening the market’s capacity to absorb policy-related shocks [60]. This is reflected in improved resource flows toward high-quality firms, more diversified financing channels that ease cash flow constraints, and stronger liquidity-driven price buffering mechanisms in the face of TPU [61, 62]. Taken together, these mechanisms suggest that marketization effectively attenuates the transmission of TPU into crash risk. Accordingly, we propose the following hypothesis:

Hypothesis 2: All else equal, a higher level of marketization mitigates the stock price crash risk induced by trade policy uncertainty.

#### 2.3.2. Improving digital transform.

Digital transformation has emerged as a critical force reshaping firm-level operating logic and market mechanisms, and is increasingly recognized as a key mitigating factor in the face of external uncertainty shocks [63–66]. To examine its moderating role in the relationship between trade policy uncertainty (TPU) and stock price crash risk, this paper analyzes three primary channels: improvements in information efficiency, enhanced organizational resilience, and the optimization of market structure.

First, digitalization strengthens the information production–transmission–absorption chain, thereby alleviating market misperceptions and price distortions induced by TPU, ultimately reducing the likelihood of crashes [67, 68]. Specifically, it enhances the timeliness and completeness of corporate disclosures, limiting managerial discretion to conceal adverse news under high TPU and mitigating the abrupt release of accumulated bad news [23]. It also empowers analysts and financial media to more effectively detect and interpret policy signals, curbing investor mis-reaction and excessive volatility [69]. Furthermore, by lowering the cost of information acquisition and reducing cognitive frictions, digital transformation improves the alignment of investor beliefs, dampening herd-driven, irrational trading behavior [69, 70].

Second, digitalization strengthens firms’ capacity to respond to shocks and perceive risks in real time, thereby enhancing internal resilience and insulating valuation from TPU-induced volatility [64]. On one hand, it improves firms’ dynamic flexibility in reconfiguring resources and adjusting supply chains to adapt to external disruptions [71]. On the other, real-time data facilitate more transparent and responsive top-level decision-making, narrowing the gap between strategic action and investor expectations [63]. Additionally, intelligent risk monitoring systems [72] enable early identification and preemptive responses to TPU-related shocks, reducing the risk of nonlinear price corrections stemming from delayed adaptation [69].

Finally, at the market level, digitalization improves the responsiveness and integration of trading mechanisms, enhancing the financial system’s capacity to absorb shocks and price TPU efficiently [66]. First, it increases the processing speed of matching systems, shortening the latency between policy announcements and price adjustments, thereby preventing abrupt price corrections caused by delayed reactions [66]. Second, it deepens and broadens market participation, ensuring stable liquidity provision even under heightened TPU [73]. Third, it refines the transaction connectivity among investors [74, 75], reducing the probability that localized panic transforms into systemic liquidity risk, thereby reinforcing market stability and resilience [74].

Hypothesis 3: Ceteris paribus, firms with higher levels of digital transformation are less vulnerable to stock price crash risk induced by trade policy uncertainty.

## 3. Data and methodology

### 3.1 Data and sample selection

We calculate the variable—trade policy uncertainty—based on annual reports of the listed firms. The financial data of enterprises are obtained from CSMAR for listed companies. The monthly index of trade policy uncertainty in China is obtained from the website http://www.policyuncer-tainty.com constructed by Baker and Wurgler [3]. To alleviate the impact of special industries, outliers, and missing values on the empirical results, a rigorous step was followed for the selection of samples and observations, and they are as follows:

1. Observations with missing values were removed.
2. The data was winsorized at the 1% level at both the upper and lower tails.

Finally, we get an unbalanced panel dataset for a period from 2001 to 2023. The software used for data analysis is Stata 18, employing the xtabond2 and reghdfe commands.

### 3.2. Variables

#### 3.2.1. Independent variable: Trade policy uncertainty.

This study is closely related to the work of Yang [76] and Benguria et al. [31]. To construct a firm-level, time-varying measure of subjective perception of trade policy uncertainty (TPU), we adopt the textual analysis approach proposed by Caldara et al. [28] and Sentiment tendency scoring method based on BosonNLP Chinese sentiment dictionary [51]. Table 1 presents the keyword lists used to identify uncertainty and trade policy references. These keywords are selected in strict accordance with the methodology outlined in Benguria et al. [31], with minor deviations attributable primarily to structural differences between Chinese and English writing conventions. The construction of our index proceeds in four steps.

**Table 1:** The lists of policy-related and uncertainty-related key words.

Type of keywords	Keywords
Policy-related words	贸易(Trade) ; 经贸(Economic and trade) ; 自贸(Free trade) ; 世贸(World Trade); 出口(Export) ; 进口(Import) ; 关税(Tariff) ; 壁垒(barriers) ; 反倾销(Anti-dumping); 外包(Outsourcing) ; 保护主义(protectionism) ; 单边主义(unilateralism).
Uncertainty-related words	不确定(uncertain) ; 不明确(unclear) ; 不明朗(unclear) ; 未明(unknown) ; 难料(unpredictable) ; 难以估计(difficult to estimate) ; 难以预计(difficult to predict) ; 难以预测(unpredictable) ; 难以预料(unpredictable) ; 风险(risk) ; 危险(danger) ; 危机(crisis) ; 威胁(threat) ; 未知(unknown).

First, we compile the textual data. Using Python, we extract annual reports of all A-share listed firms in China from 2001 to 2023 via the CNINFO platform. The collected reports are converted into.txt format and re-imported into Python for analysis.

Second, we identify uncertainty-related expressions. This step involves scanning each line of the text for words denoting uncertainty, such as “uncertainty” and “unclear” in Table 1, regardless of whether they are directly linked to trade policy.

Table 1 reports the set of keywords related to trade policy uncertainty employed in the textual analysis for constructing the STPU index. The selection of these terms closely follows the methodology outlined in Benguria et al.[31]. Minor deviations arise primarily from structural and linguistic differences between Chinese and English writing conventions.

Third, we determine co-occurrence. If an uncertainty-related word is detected, we then examine a surrounding window of 20 words—20 preceding and 20 following—to check for the presence of trade policy keywords, such as “trade,” “economic and trade,” or “free trade” in Table 1. If both uncertainty and trade-related terms are found within this window, the sentence is recorded as a valid instance of TPU co-occurrence.

Fourth, we incorporate sentiment classification. Building on the co-occurrence foundation, we construct a measure of the trade policy uncertainty (TPU) by applying a sentiment scoring algorithm based on the BosonNLP Chinese sentiment lexicon [51]. Specifically, we first segment the co-occurring TPU sentences into individual words. We then match these against the lexicon to retrieve polarity (positive or negative) and intensity scores for each emotional term. These scores are adjusted for the presence of negations (e.g., “not,” “never”) and degree adverbs (e.g., “very,” “slightly”). The total sentiment score for each sentence is obtained by summing across all emotional terms. Sentences with scores > 0 are classified as positive, those with scores < 0 as negative, and scores ≈ 0 as neutral. The final TPUT index is defined as the number of negatively scored TPU sentences in each firm-year observation. This indicator captures not only whether firms perceive trade uncertainty, but also the extent to which such uncertainty is framed in affective or anxiety-laden language—thereby offering a more behaviorally salient measure of policy-induced risk. For empirical implementation, we normalize this measure using z-score transformation at the firm level using the equation $\frac{X-\overset{―}{\overset{―}{X}}}{\sigma }$. This normalization facilitates comparability and mitigates scale effects in our regression models.

Formally, the s trade policy uncertainty in firm i in year t is calculated as follows:


$${TPU}_{i,t}=\frac{\text{1}\text{0}\text{0}}{R_{i,t}}\sum_{w=\text{1}}^{R_{i,t}}\left\{\text{1}[w\in {Keywords}^{TradePolicy}]\times \text{1}[|w-r|\leq Oneline]\right\}$$ (1)

In this equation, w represents the number of words in firm i’s annual report in year t; $R_{\text{i},\text{t}}$ denotes the total number of words in the annual report; The variable r refers to the keyword related to uncertainty closest to w, where r ∈ Keywords Uncertainty. The sentiment classification of the sentence containing keyword w is captured by the function e(w), which assigns the sentence to one of three categories—negative, neutral, or positive—based on a lexicon-based sentiment scoring algorithm. To ensure robustness, we refine the third step of the TPU construction by varying the co-occurrence window used to identify trade policy uncertainty expressions. Specifically, we compute the frequency with which uncertainty and trade-related keywords co-occur within a window of 15 and 30 characters before or after the target word w. All other aspects of the methodology remain unchanged. These alternative indices, derived from different window lengths, function as robustness checks to assess the consistency and validity of the baseline TPUT measure.

**Fig 3:**
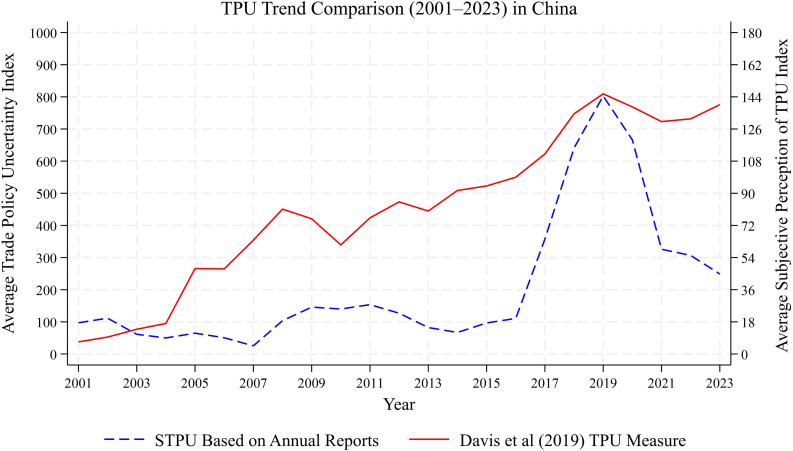
Aggregate TPUT_20 based on annual reports and TPU index by Davis et al. [2].

To assess the validity of our constructed TPU measure as a reflection of firms’ trade policy uncertainty, we compare it against the TPU index developed by Davis et al. [2], which is based on textual analysis of two major mainland Chinese newspapers. To facilitate comparison, we aggregate the firm-level STPU series into a national-level index using a weighted average approach and present the results in Fig 3. The two indices exhibit broadly similar trends over time, suggesting a high degree of co-movement. However, during periods of heightened uncertainty—most notably from 2018 to 2020—the TPU index displays substantially greater volatility. This divergence indicates that firm-based TPU captures a more pronounced sensitivity to uncertainty shocks, offering higher informational content and a stronger signal of sentiment than the newspaper-based measure.

Further, we adopt the trade policy uncertainty index (TPUI) grounded in the work of Davis et al. [2] in robustness checks. The index quantifies uncertainty-related concepts since 2000 using two mainland Chinese newspapers, People’s Daily and Guangming Daily. To capture broader trends, the monthly China TPU Index is aggregated into an annual index by applying a share-weighted method, and the natural logarithm of the annual index was calculated.

#### 3.2.2. Dependent variable: Stock price crash risk.

This section provides a description of our dependent variables proxying stock price crash risk. Stock price crash risk is defined as the likelihood of extremely negative firm-specific returns [11, 13, 15] explained by idiosyncratic factors, with the more common represented by the sudden release of bad news previously withheld by managers [38]. Based on the existing literature, we use 2 methods to measure stock price crash risk, respectively: the negative coefficient of skewness of firm-specific daily returns (NCSKEW) [11] and the crash likelihood measure of the Down-to-Up Volatility (DUVOL) of firm-specific daily returns [15].

The estimation of firm-specific stock returns utilizes an enhanced market model incorporating both lagged and lead terms for market returns, as follows:


$$r_{i,t}=\alpha +\beta_{\text{1},i}r_{m,t-\text{2}}+\beta_{\text{2},i}r_{m,t-\text{1}}+\beta_{\text{3},i}r_{m,t}+\beta_{\text{4},i}r_{m,t+\text{1}}+\beta_{\text{5},i}r_{m,t+\text{2}}+\varepsilon_{i,t}$$ (2)

Where $r_{i,t}(r_{m,t})$ is the return for stock i (stock index m) in week *t*. Then, the firm-specific weekly returns $R_{i,t}$ are derived from the residual $\varepsilon_{i,t}$ from Eq (3).


$$R_{i,t}=In(\text{1}+\varepsilon_{i,t})$$ (3)

Our main measure of crash risk is the negative coefficient of skewness (${\text{NCSKEW}}_{i,T}$), defined as the third moment of firm-specific returns:


$${NCSKEW}_{i,T}=-\frac{n(n-\text{1}{)}^{\frac{\text{3}}{\text{2}}}\sum_{i=\text{1}}^{n}R_{i,t}^{\text{3}}}{(n-\text{1})(n-\text{2}){\left(\sum_{i=\text{1}}^{n}R_{i,t}^{\text{2}}\right)}^{\frac{\text{3}}{\text{2}}}}$$ (4)

Where *n* is the number of available weekly returns for stock *i* in fiscal year *T.* The denominator is a normalization factor which allows to compare stocks with different price volatilizes. A higher value of ${\text{NCSKEW}}_{i,T}$ indicates a more pronounced negative skewness in returns, signifying a greater degree of stock price crashes. Conversely, a lower value suggests a reduced likelihood of such crashes.

The second measure of crash risk is the down-to-up volatility (${\text{DUVOL}}_{i,T}$), computed in the following way:


$${DUVOL}_{i,T}=In\left(\frac{(n_{up}-\text{1})\sum_{down}R_{i,t}^{\text{2}}}{(n_{down}-\text{1})\sum_{up}R_{i,t}^{\text{2}}}\right)$$ (5)

Where ${\text{n}}_{\text{up}}$(${\text{n}}_{\text{down}})$ represents the number of weeks during which firm *i’s* stock-specific return ${\text{R}}_{\text{i},\text{t}}$ exceeds (or falls below) the annual average return, a larger *DUVOL* value indicates a more negatively skewed return distribution, signifying a higher degree of stock price crash risk. Conversely, a smaller *DUVOL* value reflects a lower likelihood of such crashes.

#### 3.2.3. Control variables.

To ensure consistency with existing literature, we incorporate a range of control variables that may influence stock price crash risk. SN denotes the natural logarithm of the total number of shareholders, while BM is the ratio of book value to market capitalization. MFB is a binary indicator reflecting whether any current board member or senior executive has a financial background, such as prior experience in regulatory agencies, banks, securities firms, futures companies, investment institutions, or trusts. MOB captures whether any such individual has an international background, including previous or current overseas education or employment. ROE is measured as net profit divided by the average balance of owners’ equity. Size is defined as the natural logarithm of total assets, and Lev represents the ratio of total liabilities to total assets at the end of the fiscal year. Ret represents the annual average of weekly stock returns. Sigma is the standard deviation of the company’s annual weekly return rate. At the regional level, CPI and GDP refer to the provincial Consumer Price Index and the logarithm of Gross Domestic Product, respectively. A summary of all core variables is presented in Table 2, with descriptive statistics reported in Table 3.

**Table 2:** Variable definition, measurement and data source.

Variables	Definition and measurement	Data Source
TPUT	Trade policy uncertainty based on a textual analysis at the firm level, calculated from the text of annual reports.	Obtained through textual analysis
TPUI	We use the monthly China TPU Index [2], available at http://www.policyuncertainty.com. The monthly China TPU Index is aggregated into an annual index by applying a weighted average method, and the natural logarithm of the annual index was calculated.	https://www.policyuncertainty.com
NCSKEW	The negative coefficient of skewness, defined as the ratio of the third moment of firm-specific weekly returns to the cube standard deviation, multiplied by minus one [11].	Variable data or original data for variable calculations is from CSMAR.
DUVOL	The down-to-up volatility, defined as the natural logarithm of the ratio of the standard deviation of weekly returns in the “down weeks” to the standard deviation of weekly returns in the “up weeks”. Up (down) weeks refer to weeks when the firm-specific stock return is above (below) fiscal year average [15]	
SN	Natural logarithm of total shareholders	
BM	Book value/ market capitalization	
MFB	a binary indicator reflecting whether any current board member or senior executive has a financial background,	
MOB	a binary indicator reflecting whether any such individual has an international background	
ROE	Net profit/ the average balance of owner’s equity.	
Lev	Ratio of total liabilities to total assets at year-end	
Size	Natural logarithm of total assets for the year	
Ret	Annual average weekly return on stocks	
Sigma	The standard deviation of the company’s annual weekly return rate	
GDP	Natural logarithm of the gross domestic product of the province where the company is located	
CPI	Consumer price index of the province in which the company is located	

This table provides a description of the variables used in the empirical analysis.

**Table 3:** Descriptive statistics.

	(1)	(2)	(3)	(4)	(5)
VARIABLES	N	mean	sd	min	max
NCSKEW	54,273	−0.288	0.726	−2.769	2.408
DUVOL	54,273	−0.189	0.483	−1.547	1.531
TPUT_20	54,273	0.129	0.266	0.000	4.219
market	54,273	9.386	2.165	−0.161	13.356
DIG1	54,273	1.242	1.407	0.000	6.380
MFB	54,273	0.533	0.499	0.000	1.000
MOB	54,273	0.444	0.497	0.000	1.000
Size	54,273	22.109	1.507	11.348	31.431
Lev	54,273	0.488	3.930	0.002	87.256
ROE	53,403	0.078	6.207	−174.895	13.551
SN	52,282	10.369	0.939	7.477	14.517
Ret	54,273	0.003	0.011	−0.031	0.068
Sigma	54,273	0.063	0.025	0.019	0.232
CPI	54,273	4.625	0.014	4.597	4.662
GDP	54,273	10.973	0.779	8.006	12.207

This table provides a description of the variables used in the empirical analysis.

### 3.3. Empirical model and methods

According to the theory of information asymmetry [17], heightened external policy uncertainty may induce corporate managers to withhold adverse information, leading to the accumulation of undisclosed risks that are ultimately released in a concentrated manner, thereby triggering stock price crashes. From the perspective of behavioral finance, investors tend to exhibit excessive reactions and herd behavior in response to trade policy shocks, amplifying market volatility and intensifying downside tail risks [77]. Together, these theories offer a robust micro-foundation for understanding how trade policy uncertainty contributes to stock price crash risk, thus informing the theoretical framework underpinning this study.


CRASH=F(TPU,CONTROL) (6)

Where TPU is trade policy uncertainty, CRASH is the stock price crash risk, and CONTROL represents the other variables affecting the firm’s stock price crash risk. The expanded model is presented as follows:


CRASH=f(TPU,SN,BM,MFB,MOB,ROE,Lev,Size,GDP,CPI) (7)

Where SN, BM, MFB, MOB, ROE, Lev, Size, GDP, CPI are number of the shareholders, managers’ financial background, managers’ oversea background, return on equity, debt-to-asset ratio, enterprise size, CPI and GDP of local province. Subsequently, transforming the estimated model into its logarithmic form, it can be rewritten as follows:


$$\begin{array}{cc} {CRASH}_{i,t}= & \varphi_{\text{1}}+\varphi_{\text{2}}{TPU}_{i,t}+\varphi_{\text{3}}{SN}_{i,t}+\varphi_{\text{4}}{BM}_{i,t}+\varphi_{\text{5}}{MFB}_{i,t}+\varphi_{\text{6}}{MOB}_{i,t}+{\varphi_{\text{7}}ROE}_{i,t}\\ & +\varphi_{\text{8}}{Lev}_{i,t}+\varphi_{\text{9}}{GDP}_{j,t}+\varphi_{\text{1}\text{0}}{CPI}_{j,t}+\varepsilon_{i,t} \end{array}$$ (8)

Where $\varepsilon_{i,t}$ stands for error terms, *i* is the cross-section, and *t* is time. In this study, we used *t*he GMM model to estimate the above equation as it provides more information and provides less collinearity between variables [78]. The GMM model is characterized by the presence of lagged dependent variables on the right-hand side as follows:


$$\begin{array}{cc} {CRASH}_{i,t}= & \varphi_{\text{1}}+{\gamma {CRASH}_{i,t-\text{1}}+\varphi }_{\text{2}}{TPU}_{i,t}+\varphi_{\text{3}}{SN}_{i,t}+\varphi_{\text{4}}{BM}_{i,t}+\varphi_{\text{5}}{MFB}_{i,t}+\varphi_{\text{6}}{MOB}_{i,t}+{\varphi_{\text{7}}ROE}_{i,t}\\ & +\varphi_{\text{8}}{Lev}_{i,t}+\varphi_{\text{9}}{GDP}_{j,t}+\varphi_{\text{1}\text{0}}{CPI}_{j,t}+\varepsilon_{i,t} \end{array}$$ (9)

Where γ is the coefficient of the lagged dependent variable, and we will focus on the coefficient of $\varphi_{\text{2}}.$ In order to investigate the moderating marketization and digital transform on the relationship between TPU and stock price crash risk, this study gives the following estimating model.


$$\begin{array}{cc} {CRASH}_{i,t}= & \varphi_{\text{1}}+{\gamma {CRASH}_{i,t-\text{1}}+\varphi }_{\text{2}}{TPU}_{i,t}+\rho {MF}_{i,t}\times {TPU}_{i,t}+\rho_{\text{1}}{MF}_{it}+\varphi_{\text{3}}{SN}_{i,t}+\varphi_{\text{4}}{BM}_{i,t}+\varphi_{\text{5}}{MFB}_{i,t}+\varphi_{\text{6}}{MOB}_{i,t}\\ & +{\varphi_{\text{7}}ROE}_{i,t}+\varphi_{\text{8}}{Lev}_{i,t}+\varphi_{\text{9}}{GDP}_{j,t}+\varphi_{\text{1}\text{0}}{CPI}_{j,t}+\varepsilon_{i,t} \end{array}$$ (10)

Where ρ is the interesting, and here we expect it will be negatively significant.

### 3.4. Estimation technique

As the pronounced path dependence and temporal dynamics of stock price crash risk, this study employs the Generalized Method of Moments (GMM) for model estimation [79, 80]. Unlike conventional fixed effects or OLS estimators, GMM accommodates the inclusion of lagged dependent variables as regressors, thereby capturing the evolution of financial risk over time. Moreover, the potential bidirectional causality between trade policy uncertainty (TPU) and crash risk—wherein firms facing extreme downside risk may retrospectively influence managerial disclosures of uncertainty—raises endogeneity concerns. GMM addresses this by using lagged endogenous variables as internal instruments, mitigating bias arising from the correlation between explanatory variables and the error term. Taken together, the method offers strong theoretical coherence, robustness to dynamic panel structures, and improved identification of the causal impact of TPU on downside tail risk.

## 4. Empirical results

### 4.1. Benchmark regression

This study examines the hypothesis that Trade Policy Uncertainty (TPU) significantly increases stock price crash risk. A firm-level measure of TPU (TPUT) is used as the core explanatory variable, while two widely adopted proxies for crash risk—negative conditional skewness (NCSKEW) and down-to-up volatility (DUVOL)—serve as dependent variables in the regression analysis (see Table 4 for details). To address endogeneity concerns and improve estimation robustness, dynamic panel models are constructed following Equation (9). The regression results (Columns 1–8) reveal a statistically significant positive association between TPUT and both crash risk indicators, suggesting that rising trade policy uncertainty exacerbates trading frictions, which in turn triggers the concentrated release of downside risk, thereby increasing the probability of stock price crashes. These findings offer empirical validation for Hypothesis 1.

**Table 4:** Baseline regressions.

	(1)	(2)	(3)	(4)	(5)	(6)	(7)	(8)
	Two-step Sys-gmm	Two-step Diff-gmm	Two-step Sys-gmm	Two-step Diff-gmm	Two-step Sys-gmm	Two-step Sys-gmm	Two-step Sys-gmm	Two-step Sys-gmm
VARIABLES	NCSKEW	NCSKEW	DUVOL	DUVOL	NCSKEW	DUVOL	NCSKEW	DUVOL
RISK(−1)	−0.470***	−0.429*	−0.457***	−1.205***	−0.478***	−0.455***	−0.484***	−0.720***
	[-3.176]	[-1.716]	[-3.712]	[-11.328]	[-3.106]	[-3.468]	[-2.958]	[-4.324]
TPUT_20	3.529**		1.651**					
	[2.502]		[2.091]					
TPUT_20(−1)		1.931**		0.960**				
		[2.116]		[2.520]				
TPUT_15					3.748***	1.801**		
					[2.821]	[2.179]		
TPUT_30							1.941***	1.077*
							[2.774]	[1.748]
MFB	0.537	−0.039	0.414**	0.055	0.532	0.366**	0.266	−0.110
	[1.594]	[-0.109]	[2.284]	[0.417]	[1.560]	[1.984]	[0.854]	[-0.204]
MOB	−0.240	−0.018	−0.189	−0.068	−0.321	−0.169	−0.107	0.398
	[-0.609]	[-0.047]	[-0.984]	[-0.447]	[-0.828]	[-0.927]	[-0.300]	[0.837]
Size	−0.006	0.006	0.103	−0.072	0.014	0.114	−0.403	−0.146
	[-0.032]	[0.015]	[1.037]	[-1.116]	[0.080]	[1.271]	[-1.200]	[-0.867]
Lev	0.025	0.298	0.146	−0.004	0.014	−0.063	−1.360	−2.210
	[0.352]	[0.206]	[0.535]	[-0.729]	[0.302]	[-0.221]	[-1.079]	[-1.370]
ROE	−0.001	0.035	0.022	−0.000	−0.001*	0.001	−0.187	−0.210
	[-1.243]	[0.430]	[0.412]	[-1.209]	[-1.732]	[0.011]	[-0.983]	[-1.386]
SN	−0.118	−0.261	−0.252	−0.043	−0.211	−0.229	0.418	0.296
	[-0.472]	[-0.458]	[-1.218]	[-0.537]	[-0.907]	[-1.165]	[0.806]	[0.702]
Ret	−17.710	−29.858***	−21.424**	−18.621***	−18.307	−18.212**	−13.919	−21.571*
	[-1.510]	[-3.420]	[-2.375]	[-4.552]	[-1.626]	[-2.018]	[-1.466]	[-1.824]
Sigma	3.924	5.716	0.366	2.243	3.916	−0.518	5.298	−7.043
	[0.739]	[1.571]	[0.104]	[1.155]	[0.793]	[-0.149]	[1.271]	[-1.168]
CPI	−3.758	−5.082*	−4.377	−3.510**	−3.022	−3.236	−4.344	−5.410
	[-1.011]	[-1.874]	[-1.557]	[-2.477]	[-0.816]	[-1.166]	[-1.142]	[-1.232]
GDP	−0.029	−0.083	−0.087*	−0.027	−0.023	−0.111**	0.003	−0.309*
	[-0.280]	[-0.996]	[-1.701]	[-0.800]	[-0.223]	[-2.009]	[0.025]	[-1.658]
Constant	18.662	26.243*	21.373	18.277***	15.781	16.063	24.962	30.098
	[1.073]	[1.810]	[1.612]	[2.664]	[0.917]	[1.234]	[1.258]	[1.388]
	Model Criteria							
Hansen	0.724	0.114	0.479	0.451	0.553	0.253	0.204	0.367
AR(1)	0.000***	0.012**	0.000***	0.000***	0.000***	0.000***	0.000***	0.0279**
AR(2)	0.256	0.219	0.110	0.0972*	0.229	0.158	0.227	0.116
Dif-Sar	32	13	32	36	32	32	32	28
#inst	45	26	45	49	45	45	45	41
#id	4314	4314	4314	4314	4314	4314	4314	4314

This table reports the estimated effects of trade policy uncertainty (TPU) on firms’ stock price crash risk. Columns (1) to (4) employ TPUT_20 as the firm-level measure of trade policy uncertainty, with Columns (2) and (4) using the one-period lag of TPUT_20, while Columns (5) to (8) use TPUT_15 and TPUT_30 as alternative proxies. The dependent variables are NCSKEW and DUVOL, both capturing stock price crash risk. Based on Equation (10), the model is estimated using two alternative dynamic panel estimators for robustness: the two-step system GMM and the two-step difference GMM. Z-values are clustered at the firm level and reported in brackets. *, **, and *** indicate statistical significance at the 10%, 5%, and 1% levels, respectively.

In Table 4, in the TPUT_20 – and lagged TPUT_20 -based specifications (Columns 1–4), the estimated coefficients on TPUT are consistently positive and statistically significant: 3.529 in Column 1 (5% level), 1.931 in Column 2 (1% level), 0.435 in Column 3 (1% level), and 0.96 in Column 4 (5% level). Although the lagged coefficients remain statistically significant, their magnitudes are notably smaller than the contemporaneous estimates, suggesting that TPU’s impact is most immediate but persists into subsequent periods with reduced intensity. This pattern implies that firms and investors react promptly to heightened trade policy uncertainty, while the delayed effect reflects gradual adjustments in disclosure strategies, investment behavior, and market expectations. The findings therefore support the theoretical view that policy uncertainty shocks not only trigger contemporaneous responses but also leave a lingering influence on firm-level fragility, albeit at a weaker scale in later periods.

Likewise, in the TPUT_15 – and TPUT_30 -based models (Columns 5–8), the coefficients remain positive and robust across specifications: 3.748 in Column 5 (1% level), 1.801 in Column 6 (5% level), 1.941 in Column 7 (1% level), and 1.077 in Column 8 (10% level). All the results support Hypothesis 1.

From the perspective of information asymmetry, heightened trade policy uncertainty (TPU) exacerbates the uneven distribution of firm-specific information between managers and outside investors. Under greater uncertainty, managers have stronger incentives to strategically delay or obscure the disclosure of adverse information in order to preserve market valuation or protect private interests [81] This selective disclosure intensifies the opacity of the information environment, raising investors’ difficulty in distinguishing firm fundamentals. As negative information accumulates and is eventually released in a concentrated manner, the sudden adjustment of market expectations generates sharp downward price corrections. The empirical evidence—specifically, the consistently positive and significant coefficients of TPUT on both NCSKEW and DUVOL—corroborates this information asymmetry channel, indicating that policy uncertainty magnifies crash risk by aggravating the opacity of corporate information disclosure.

### 4.2 Robustness tests

#### 4.2.1 Changing TPUT to TPUI.

This study extends the robustness analysis by substituting the key regressor with the Trade Policy Uncertainty Index (TPUI) for China, developed by Davis et al.[2], and re-estimating the effect on crash risk. Table 5, Columns (1) and (2), report the corresponding results. The estimated coefficients on TPUI are positive and statistically significant at the 10% and 5% levels, respectively, indicating that higher levels of trade policy uncertainty are associated with an elevated likelihood of stock price crashes. Specifically, TPUI increases NCSKEW by 0.145 (Column 1) and DUVOL by 0.423 (Column 2). Overall, these findings confirm the robustness of the baseline evidence and reinforce the conclusion that trade policy uncertainty systematically heightens firms’ crash risk exposure.

**Table 5:** Changing TPU’s indicator and other determinant factors.

	(1)	(2)	(3)	(4)	(5)	(6)	(7)	(8)
VARIABLES	NCSKEW	DUVOL	NCSKEW	NCSKEW	NCSKEW	NCSKEW	NCSKEW	NCSKEW
RISK (−1)	−0.019*	−0.487***	−0.550***	−0.477***	−0.645***	−0.464***	−0.444***	−0.551***
	[-1.822]	[-3.359]	[-3.287]	[-3.222]	[-4.988]	[-3.235]	[-2.941]	[-3.679]
TPUT_20			3.374**	3.442**	3.218***	3.301**	4.121**	3.404***
			[2.353]	[2.397]	[3.175]	[2.273]	[2.495]	[2.844]
TPUI	0.145*	0.423**						
	[1.673]	[2.011]						
ReportAttention			−0.019					0.018
			[-1.634]					[1.255]
Media_7				−0.198				−0.004
				[-0.969]				[-0.013]
AQ					−0.742			−2.301
					[-0.549]			[-1.116]
IndDirectorRatio						−0.036		0.065
						[-0.973]		[1.138]
Shrhfd							−3.553	14.440
							[-0.659]	[1.554]
MFB	0.205	0.061	0.750*	0.523	0.487	0.500	0.446	0.460
	[1.021]	[0.319]	[1.903]	[1.571]	[1.364]	[1.459]	[1.140]	[0.872]
MOB	−0.226	0.044	−0.378	−0.129	−0.346	−0.227	−0.178	−0.345
	[-0.831]	[0.129]	[-0.864]	[-0.306]	[-0.904]	[-0.597]	[-0.414]	[-0.577]
Size	0.804***	−0.108	0.461	0.246	0.040	0.071	−0.112	−0.216
	[3.111]	[-0.456]	[1.322]	[0.770]	[0.183]	[0.325]	[-0.461]	[-0.347]
Lev	0.007	0.000	−0.019	0.022	1.665	0.025	0.014	2.967*
	[0.566]	[0.249]	[-0.363]	[0.337]	[1.170]	[0.316]	[0.161]	[1.781]
ROE	−0.000	−0.001**	−0.000	−0.001	−0.003	−0.001	−0.001	−0.002
	[-0.314]	[-2.114]	[-0.409]	[-1.228]	[-0.248]	[-1.460]	[-1.194]	[-0.149]
SN	−0.864***	−0.863**	−0.560	−0.315	−0.508	−0.203	−0.343	0.411
	[-2.867]	[-2.253]	[-1.532]	[-1.076]	[-1.540]	[-0.744]	[-0.704]	[0.447]
Ret	−27.405***	−15.326	−17.715	−17.562	−28.787**	−20.250*	−19.172	−17.791
	[-4.156]	[-1.182]	[-1.483]	[-1.506]	[-2.350]	[-1.677]	[-1.493]	[-1.224]
Sigma	5.007*	2.787	6.656	2.241	12.256*	4.575	6.162	8.627
	[1.691]	[0.601]	[1.200]	[0.401]	[1.958]	[0.846]	[1.089]	[1.244]
CPI	−5.519**	4.486	−3.016	−2.294	−6.999*	−4.313	−3.708	−6.679
	[-2.530]	[0.442]	[-0.777]	[-0.561]	[-1.674]	[-1.170]	[-0.931]	[-1.095]
GDP	−0.523***	0.770	−0.206	−0.004	0.013	−0.021	−0.029	0.363
	[-3.744]	[1.564]	[-1.287]	[-0.038]	[0.131]	[-0.183]	[-0.163]	[1.400]
Constant	21.914**	−17.938	11.478	9.052	35.086*	21.631	23.537	20.624
	[2.228]	[-0.380]	[0.608]	[0.432]	[1.761]	[1.257]	[1.232]	[0.669]
	Model Criteria							
Hansen	0.147	0.488	0.817	0.845	0.144	0.705	0.860	0.270
AR(1)	0.000***	0.004***	0.000***	0.000***	0.000***	0.000***	0.000***	0.000***
AR(2)	0.150	0.169	0.136	0.252	0.0114	0.245	0.444	0.0518
Dif-Sar	35	32	31	31	31	31	31	27
#inst	48	45	45	45	45	45	45	45
#id	4770	4314	4309	4242	4094	4314	4314	4046

This table reports robustness tests using alternative measures of trade policy uncertainty as well as other important factors that may influence the relationship between TPU and stock price crash risk. Columns (1) and (2) employ the TPUI constructed by Davis et al. [2] as a proxy for TPU. Columns (3) to (7) sequentially introduce analyst attention, media coverage, audit quality, the proportion of independent directors, and ownership concentration to assess their effects on the relationship. Column (8) incorporates all these factors jointly into a single regression. Estimation is conducted using the two-step system GMM approach. Z-values are clustered at the firm level and reported in brackets. *, **, and *** indicate statistical significance at the 10%, 5%, and 1% levels, respectively.

#### 4.2.2 Other determinant factors.

Building on the baseline specification, Columns (3) to (7) sequentially introduce additional determinants that may shape the link between trade policy uncertainty and crash risk in Table 5. Specifically, analyst attention, media coverage, audit quality, the proportion of independent directors, and ownership concentration are each incorporated in turn. Across all specifications, the coefficients on trade policy uncertainty remain positive and significant, indicating that the destabilizing effect of policy uncertainty on crash risk is robust and not driven by any single firm-level characteristic. Column (8) further includes all these determinants simultaneously, and the results continue to confirm a strong and consistent association between trade policy uncertainty and stock price crash risk.

#### 4.2.3 Subsample analysis on industries.

Table 6 reports the subsample results across different industries to examine the heterogeneity in the impact of trade policy uncertainty on stock price crash risk. Column (1) focuses on high-tech firms, Column (2) on manufacturing firms, Column (3) on property developers, and Column (4) on financial institutions. Across all four subsamples, the coefficients on TPUT_20 remain positive and statistically significant, suggesting that heightened trade policy uncertainty systematically increases the likelihood of stock price crashes irrespective of industry type. Overall, these findings confirm that the destabilizing role of trade policy uncertainty is robust across industries, though its intensity varies depending on sectoral characteristics.

**Table 6:** Sub-sample analysis.

	(1)	(2)	(3)	(4)
Industry	High-tech	manufactory	Property	Finance
	Two-step Sys-gmm	Two-step Sys-gmm	Two-step Sys-gmm	Two-step Sys-gmm
VARIABLES	NCSKEW	NCSKEW	NCSKEW	NCSKEW
L.NCSKEW	−0.269**	−0.380***	−0.528***	−0.223
	[-2.154]	[-2.726]	[-5.249]	[-1.577]
TPUT_20	2.391**	2.203*	3.518***	0.997**
	[2.392]	[1.716]	[2.665]	[2.261]
MFB	0.213	0.164	0.031	0.486
	[0.624]	[0.382]	[0.155]	[1.383]
MOB	0.001	0.068	0.121	−0.351
	[0.004]	[0.177]	[0.462]	[-0.753]
Size	−0.006	0.114	−0.013	−0.324*
	[-0.033]	[0.548]	[-0.256]	[-1.805]
Lev	−0.006	−0.168	0.039**	1.519
	[-0.307]	[-0.361]	[1.985]	[0.923]
ROE	−0.018	−0.005	0.024	0.188
	[-1.416]	[-0.532]	[0.150]	[0.421]
SN	−0.093	−0.192	−0.301*	0.094
	[-0.396]	[-0.853]	[-1.879]	[0.271]
Ret	−21.225	−22.837	−10.454**	−10.631**
	[-1.436]	[-1.591]	[-2.533]	[-2.140]
Sigma	4.703	2.354	4.779**	−9.355**
	[0.643]	[0.345]	[2.402]	[-2.192]
CPI	−4.206	−4.633	0.760	−1.176
	[-0.889]	[-0.994]	[0.316]	[-0.353]
GDP	−0.045	−0.115	0.098	0.336*
	[-0.485]	[-0.988]	[1.406]	[1.702]
Constant	20.692	22.067	−1.466	7.492
	[0.928]	[1.004]	[-0.126]	[0.483]
	Model Criteria			
Hansen	0.580	0.203	0.621	0.179
AR(1)	0.000***	0.000***	0.000***	0.011**
AR(2)	0.893	0.409	0.222	0.109
Dif-Sar	32	32	57	36
#inst	45	45	70	49
#id	3064	2810	172	126

The table reports subsample regressions by industry. Column (1) corresponds to high-tech firms, Column (2) to manufacturing firms, Column (3) to property firms, and Column (4) to financial institutions. The key explanatory variable is TPUT_20, and the dependent variable is NCSKEW. Estimation is conducted using the two-step system GMM approach, with Z-values clustered at the firm level and reported in brackets. Symbols *, **, and *** denote statistical significance at the 10%, 5%, and 1% levels, respectively.

#### 4.2.4. Endogeneity analysis.

Although the baseline estimations rely on dynamic panel techniques such as GMM to mitigate endogeneity, concerns of reverse causality remain—specifically, the possibility that anticipated market volatility may feed back into firms’ subjective disclosure of uncertainty in annual reports. This study adopts the U.S. Trade Policy Uncertainty Index (TPU_US) as an external instrument for firm-level TPU in China. The choice of TPU_US is grounded in both exogeneity and relevance. On the exogeneity side, the index is constructed from U.S. policy-related news texts [7] and is unlikely to be influenced by firm-level characteristics of Chinese listed companies. On the relevance side, a growing body of research documents the international spillover effects of U.S. trade policy uncertainty, which directly shape China’s external trade environment and corporate strategies. For example, Yan et al. [82] shows that fluctuations in U.S. TPU transmit to China’s exports and investment, while Suwanprasert [83] demonstrates that U.S. TPU shocks generate measurable spillover effects on foreign economies. At the micro level, Gao and Zhou [84] highlight that firms embedded in global supply chains are particularly exposed to U.S. policy uncertainty. Collectively, these findings confirm that U.S. TPU systematically influences Chinese firms’ trade prospects and disclosure behaviors, thereby serving as a theoretically valid and empirically relevant instrument for identifying the causal effect of TPU.

Based on this, we implement an instrumental variable strategy using two-stage least squares (2SLS). In the first stage, TPU_US serves as the instrument to predict firm-level TPU (TPUT), capturing exogenous global policy uncertainty shocks that are plausibly orthogonal to firm-specific attributes in China. In the second stage, the fitted values of firm-level TPU (TPUT_hat) are used to estimate their effect on stock price crash risk. This approach, reported in Table 7, provides more rigorous identification of the causal link between trade policy uncertainty and firm-level crash risk.

**Table 7:** Endogenous analysis.

	(1)	(2)	(3)
	First Stage	Second Stage	
VARIABLES	TPUT_20	NCSKEW	DUVOL
TPU_US	0.007***		
	[6.62]		
TPUT_hat		3.320***	3.981***
		[4.58]	[11.34]
MFB	−0.000	0.038***	0.023***
	[-0.22]	[3.60]	[4.33]
MOB	−0.005**	0.012	0.020***
	[-2.02]	[1.09]	[3.45]
Size	−0.009***	0.022**	0.024***
	[-4.94]	[2.10]	[4.80]
Lev	−0.000	−0.001**	−0.000
	[-0.90]	[-2.07]	[-0.43]
ROE	−0.000	0.000***	0.001***
	[-0.34]	[3.42]	[7.12]
SN	−0.002	0.111***	0.071***
	[-0.76]	[11.60]	[14.44]
Ret	−0.223**	14.122***	11.274***
	[-2.12]	[28.06]	[42.96]
Sigma	0.083*	0.691***	1.121***
	[1.74]	[2.98]	[9.81]
CPI	−0.307***	1.823***	1.618***
	[-3.82]	[4.93]	[8.95]
GDP	0.047***	−0.121***	−0.134***
	[16.72]	[-3.12]	[-7.07]
Constant	1.243***	−8.933***	−7.749***
	[3.24]	[-5.30]	[-9.29]
	Model Criteria		
Observations	51,087	43,772	51,088
R-squared	0.522	0.151	0.189
Firm FE	YES	YES	YES
industry FE	YES	YES	YES
Anderson canon. corr. LM statistic: Chi-sq(1)= 39.97, P-val = 0.0000			
Anderson-Rubin Wald test: F(1,39130)=21.88, P-val = 0.0000			
Cragg-Donald test (Weak-identification): 35.76 > 16.38			

This table presents the results of the endogeneity analysis, in which U.S. trade policy uncertainty index is employed as an instrumental variable. Column (1) reports the first-stage regression outcomes, where the dependent variable is firm-level trade policy uncertainty (TPUT_20). Columns (2) through (3) display the second-stage regression estimates, with the dependent variables being NCSKEW and DUVOL respectively. T-values are clustered at the firm level and reported in brackets. *, **, and *** indicate statistical significance at the 10%, 5%, and 1% levels, respectively.

Empirical findings demonstrate that in the first-stage regression, the coefficient on TPU_US is 0.07 and statistically significant at the 1% level, indicating strong instrument relevance. In the second stage, TPUT_hat is positively and significantly associated with both NCSKEW (coefficient = 3.320) and DUVOL (coefficient = 3.981), each significant at the 1% level. The Anderson canon. corr. LM statistic confirms the presence of endogeneity at the 1% level, validating the need for an IV approach. Additionally, the Anderson-Rubin Wald test yields a p-value of 0.000, and the Cragg–Donald F-statistic reaches 35.76, well above conventional thresholds 16.83, thereby mitigating concerns of under-identification and weak instrument bias. These results lend further support to the claim that trade policy uncertainty significantly amplifies the risk of stock price crashes, consistent with prior findings by Baker et al. [7], and enhance the overall credibility and robustness of the paper’s conclusions.

### 4.3 Heterogeneity analysis

To further explore how firm characteristics shape the relationship between trade policy uncertainty and stock price crash risk, Table 8 presents heterogeneity analyses along four dimensions: ownership structure, CEO duality, internationalization, and auditing by the Big Four.

**Table 8:** Heterogeneous analysis.

	(1)	(2)	(3)	(4)	(5)	(6)	(7)	(8)
	Two-step Sys-gmm	Two-step Sys-gmm	Two-step Sys-gmm	Two-step Sys-gmm	Two-step Sys-gmm	Two-step Sys-gmm	Two-step Sys-gmm	Two-step Sys-gmm
VARIABLES	NCSKEW	NCSKEW	NCSKEW	NCSKEW	NCSKEW	NCSKEW	NCSKEW	NCSKEW
	SOE = 1	SEO = 0	Dual = 0	Dual = 1	Inter = 1	Inter = 0	Big4 = 1	Big4 = 0
L.NCSKEW	−0.398**	−0.367***	0.047	−0.144***	−0.473***	−0.373***	−0.412***	−0.559***
	[-2.265]	[-3.470]	[0.296]	[-2.767]	[-3.764]	[-3.726]	[-4.677]	[-3.594]
TPUT_20	0.728	3.000***	0.607	1.494**	1.462*	−0.044	−0.929	1.753**
	[0.671]	[3.471]	[0.508]	[2.181]	[1.790]	[-1.329]	[-1.136]	[1.995]
MFB	0.451	−0.200	0.453**	0.493***	0.364	0.327	−0.091	−0.387
	[0.851]	[-0.709]	[2.361]	[2.607]	[0.813]	[1.615]	[-0.344]	[-1.412]
MOB	−0.449	0.389	−0.784*	−0.396**	−0.170	−0.163	0.234	0.508*
	[-0.571]	[1.062]	[-1.795]	[-1.982]	[-0.406]	[-0.675]	[0.796]	[1.652]
Size	−0.190	−0.216*	0.441**	0.155	0.388*	0.053	−0.793**	0.160
	[-0.477]	[-1.787]	[2.164]	[1.140]	[1.667]	[0.404]	[-2.461]	[1.145]
Lev	1.461	−0.002	−0.049	0.004**	0.079	−0.001	0.209	0.048
	[0.858]	[-1.411]	[-0.271]	[2.311]	[0.140]	[-0.092]	[0.111]	[0.924]
ROE	−0.016	0.037*	0.000	0.080	−0.021***	−0.001***	0.601	−0.054
	[-0.559]	[1.717]	[0.045]	[0.994]	[-2.689]	[-3.984]	[0.685]	[-0.382]
SN	−0.264	−0.084	−0.546	0.019	−0.466*	−0.232	0.112	−0.433*
	[-0.503]	[-0.508]	[-1.065]	[0.116]	[-1.787]	[-0.870]	[0.393]	[-1.958]
Ret	−21.822	−28.701***	−10.817	−17.015***	−15.897	−33.598***	−47.668***	−36.764***
	[-0.527]	[-3.758]	[-0.805]	[-7.490]	[-1.579]	[-3.199]	[-6.060]	[-4.103]
Sigma	5.470	10.258***	−2.782	4.714***	5.903	0.742	4.208*	7.991
	[0.727]	[2.736]	[-0.370]	[2.972]	[1.144]	[0.180]	[1.764]	[1.611]
CPI	−2.977	−8.809***	1.723	−2.233**	−4.423	−5.605	−10.970***	−7.192**
	[-0.220]	[-3.176]	[0.301]	[-2.095]	[-1.366]	[-1.451]	[-3.819]	[-2.329]
GDP	0.215	0.036	−0.262*	−0.117*	−0.150*	−0.116*	0.758*	−0.145*
	[0.546]	[0.500]	[-1.927]	[-1.945]	[-1.657]	[-1.824]	[1.889]	[-1.819]
Constant	17.476	45.275***	−9.162	7.372	17.776	28.400	60.077***	35.409**
	[0.275]	[3.386]	[-0.372]	[1.325]	[1.096]	[1.488]	[4.436]	[2.409]
	Model Criteria							
Hansen	0.462	0.279	0.949	0.438	0.336	0.481	0.164	0.111
AR(1)	0.00292	4.26e-07	0.00129	5.09e-09	0.00126	1.08e-05	2.76e-06	0.000674
AR(2)	0.340	0.294	0.995	0.160	0.124	0.144	0.165	0.0735
#inst	26	64	26	72	68	68	68	41
#id	1544	2985	3786	2752	2122	3458	452	4118

This table reports the results of the heterogeneity analysis with respect to ownership structure, CEO duality, internationalization, and auditing by the Big Four. The dependent variable is NCSKEW, and the key explanatory variable is TPUT_20. The analysis is conducted following Equation (10) and estimated using the two-step system GMM approach. Columns (1) and (2) present the results by ownership type, Columns (3) and (4) examine the role of CEO duality, Columns (5) and (6) test for the effect of internationalization, and Columns (7) and (8) analyze the impact of Big Four auditing. T-values are clustered at the firm level and reported in brackets.

Columns (1) and (2) report the results by ownership type. The positive and significant coefficients suggest that trade policy uncertainty heightens crash risk for both state-owned and non-state-owned enterprises. The effect, however, is stronger in non-state-owned firms, indicating that their relatively limited policy support and weaker capacity to buffer external shocks make them more exposed to policy-driven instability.

Columns (3) and (4) analyze CEO duality. The positive effect of TPUT_20 persists in both subsamples and is stronger when the CEO also serves as board chair. This suggests that concentrated managerial power facilitates the concealment of negative information, delaying market signals and ultimately intensifying crash risk once such information is disclosed.

Columns (5) and (6) distinguish between the internationalized and non-internationalized firms. The results indicate that the positive impact of TPUT_20 on crash risk is significantly stronger for internationalized firms. This pattern implies that globally engaged firms are more directly exposed to trade-related policy shifts, tariff volatility, and cross-border regulatory frictions, making them particularly vulnerable when policy uncertainty intensifies.

Columns (7) and (8) assess the role of external auditing quality. The effect of TPUT_20 is insignificant for firms audited by the Big Four but remains positive and significant for those audited by non-Big Four auditors. This indicates that high-quality auditing enhances transparency and reduces the transmission of policy uncertainty into crash risk, whereas weaker auditing amplifies information opacity and heightens firms’ vulnerability to uncertainty shocks.

### 4.4. Moderating effect analysis

To further investigate the channels through which institutional and organizational features condition the effect of trade policy uncertainty (TPU) on stock price crash risk, Table 9 reports the moderating roles of external marketization and internal digital transformation. Columns (1) to (4) examine marketization, while Columns (5) to (8) focus on digital transformation. The dependent variables are NCSKEW and DUVOL, estimated using the two-step System GMM and two-step Difference GMM approaches.

**Table 9:** Moderating analysis.

	(1)	(2)	(3)	(4)	(5)	(6)	(7)	(8)
	Two-step Sys-GMM	Two-step Diff-GMM	Two-step Sys-GMM	Two-step Diff-GMM	Two-step Sys-GMM	Two-step Diff-GMM	Two-step Sys-GMM	Two-step Diff-GMM
VARIABLES	NCSKEW	NCSKEW	DUVOL	DUVOL	NCSKEW	NCSKEW	DUVOL	DUVOL
RISK(−1)	−0.428***	−0.442***	0.015	0.211*	−0.003	0.162*	0.209*	−0.199
	[-4.103]	[-4.272]	[0.085]	[1.670]	[-0.024]	[1.885]	[1.741]	[-1.443]
TPU⊆mark	−1.941**	−1.860*	−1.526**	−0.922**				
	[-2.060]	[-1.867]	[-2.051]	[-2.219]				
mark	−0.147	−0.532*	0.138	0.022				
	[-0.668]	[-1.833]	[1.346]	[0.279]				
TPU⊆DIG					−1.524*	−1.447*	−1.190**	−0.065**
					[-1.686]	[-1.863]	[-2.178]	[-2.040]
DIG					0.058	0.112	0.216*	−0.015
					[0.318]	[0.961]	[1.703]	[-0.575]
TPUT_20	0.401	−0.657	0.099	−0.113	0.587	0.201	0.094	−0.039***
	[0.311]	[-0.574]	[0.128]	[-0.206]	[0.711]	[0.165]	[0.193]	[-2.659]
MFB	0.071	−0.155	−0.442*	−0.308**	0.284*	0.055	−0.132	0.038
	[0.215]	[-0.445]	[-1.847]	[-2.135]	[1.826]	[0.391]	[-1.266]	[0.307]
MOB	−0.255	−0.100	0.385**	0.349*	−0.224	0.199	0.063	0.042
	[-0.658]	[-0.247]	[2.101]	[1.894]	[-1.088]	[1.079]	[0.511]	[0.308]
Size	1.037***	0.605	0.113	−0.092	0.520***	−0.704***	0.064	−0.130
	[3.079]	[1.468]	[0.606]	[-0.629]	[2.612]	[-3.721]	[0.554]	[-1.169]
Lev	−0.014	−0.003	0.060	0.182	0.008	−0.030***	0.022*	0.014
	[-0.229]	[-0.030]	[1.110]	[1.242]	[0.384]	[-3.910]	[1.710]	[0.433]
ROE	−0.001**	−0.001**	−0.014	−0.000	−0.000	0.105*	−0.001***	0.013
	[-2.084]	[-2.122]	[-0.416]	[-1.256]	[-0.233]	[1.929]	[-3.094]	[0.552]
SN	−0.910*	−0.976**	−0.373	−0.391**	−0.415*	0.036	−0.363**	−0.206
	[-1.901]	[-1.961]	[-1.032]	[-2.185]	[-1.838]	[0.126]	[-2.256]	[-1.330]
Ret	−72.015***	−66.882***	−20.087***	−23.139***	−28.062***	−23.834***	−22.730***	−19.785***
	[-5.120]	[-4.797]	[-3.224]	[-4.375]	[-3.560]	[-5.776]	[-4.535]	[-4.517]
Sigma	17.467**	15.055**	2.299	3.031	5.471	1.170	4.311*	3.749*
	[2.532]	[2.137]	[0.468]	[1.273]	[1.406]	[0.322]	[1.715]	[1.720]
CPI	−20.494***	−21.242***	−4.124**	−6.246***	−6.294***	−6.203***	−4.322***	−5.314***
	[-4.343]	[-3.809]	[-2.300]	[-2.997]	[-2.592]	[-4.014]	[-2.858]	[-3.063]
GDP	−0.382	1.190**	−0.107	0.429**	−0.343***	0.723***	−0.125**	0.290***
	[-1.592]	[2.128]	[-0.894]	[2.024]	[-2.804]	[3.617]	[-2.238]	[3.540]
Constant	84.819***		21.432***		25.082**		23.424***	
	[4.021]		[2.595]		[2.101]		[3.108]	
	Model Criteria							
Hansen	0.493	0.599	0.815	0.290	0.664	0.0937	0.157	0.212
AR(1)	0.000***	0.000***	0.000***	0.000***	0.000***	0.026**	0.000***	0.006***
AR(2)	0.0779	0.0517	0.747	0.156	0.793	0.276	0.0701	0.139
#inst	67	63	45	64	70	45	68	66
#id	4314	3772	4770	4314	4770	4314	4770	4314
	Marginal Effect							
Min	8.044	6.668	6.109	3.518	6.944	6.240	5.058	0.230
Mean	0.401	−0.657	0.099	−0.113	0.587	0.201	0.094	−0.039
Max	−6.856	−7.613	−5.608	−3.562	−6.068	−6.120	−5.102	−0.321

This table reports the moderating effects of external marketization and internal digital transformation on the relationship between trade policy uncertainty (TPU) and stock price crash risk. Columns (1) to (4) present the results for marketization, incorporating TPUT × Mark as the core interaction term, while Columns (5) to (8) examine the role of digital transformation, employing TPUT × DIG as the core interaction term. The dependent variables are NCSKEW and DUVOL. Estimations are conducted using the two-step System GMM and the two-step Difference GMM approaches. Z-values are clustered at the firm level and reported in brackets. *, **, and *** indicate statistical significance at the 10%, 5%, and 1% levels, respectively.

Columns (1) through (4) incorporate the interaction term TPUT × Mark. The estimated coefficients on the interaction term are consistently negative and statistically significant across specifications. For instance, in Column (1) the coefficient is –1.941, while in Column (2) it remains negative at –1.860, both significant at conventional levels. Similarly, in Columns (3) and (4), the interaction terms continue to exhibit negative and significant estimates (–1.526 and –0.922, respectively). These results suggest that higher levels of marketization mitigate the destabilizing effect of TPU on stock price crash risk. In other words, a more market-oriented institutional environment provides firms with greater resilience by improving resource allocation, enhancing competition, and reducing distortions, thereby weakening the link between policy uncertainty and crash risk.

Columns (5) through (8) evaluate the moderating role of digital transformation, incorporating TPUT × DIG as the key interaction term. The estimated coefficients on the interaction term are also negative and significant across all models. Specifically, Column (5) reports –1.524, Column (6) –1.447, Column (7) –1.190, and Column (8) –0.065, with most coefficients reaching the 1% or 5% significance levels. These findings indicate that firms with deeper digital transformation are better equipped to absorb shocks from trade policy uncertainty. Enhanced digital capabilities improve information processing, operational flexibility, and strategic responsiveness, which collectively reduce firms’ exposure to extreme downside risk.

Taken together, the evidence shows that both external marketization and internal digital transformation play significant mitigating roles in the transmission of trade policy uncertainty to stock price crash risk. Marketization alleviates the adverse effect by strengthening institutional foundations and market mechanisms, while digital transformation cushions firms through enhanced technological adaptability. These results highlight the importance of institutional reforms and digital strategies in stabilizing capital markets under heightened policy uncertainty

### 4.5 Mechanism check

Table 10 reports the results of the mechanism analysis, testing several potential channels through which trade policy uncertainty (TPU) affects stock price crash risk. Columns (1) – (10) correspond to five distinct mediating mechanisms: discretionary accruals, export, investor sentiment, analyst forecast bias, and information asymmetry.

**Table 10:** Mechanism analysis.

	(1)	(2)	(3)	(4)	(5)	(6)	(7)	(8)	(9)	(10)
	Two-step Sys-GMM	Two-step Diff-GMM	Two-step Sys-GMM	Two-step Diff-GMM	Two-step Sys-GMM	Two-step Diff-GMM	Two-step Sys-GMM	Two-step Diff-GMM	Two-step Sys-GMM	Two-step Diff-GMM
VARIABLES	DA	NCSKEW	Export	NCSKEW	Sentiment	NCSKEW	FBias	NCSKEW	ASY	NCSKEW
Dv(−1)	0.011	−0.193**	−0.089	−0.161	0.552***	−0.175	−0.235	−0.372***	0.696***	0.158
	[0.206]	[-2.099]	[-1.067]	[-1.187]	[5.405]	[-1.247]	[-1.372]	[-10.070]	[8.923]	[1.488]
DA		0.083*								
		[1.838]								
Export				0.156**						
				[2.420]						
Sentiment						0.276**				
						[2.100]				
FBias								61.781**		
								[2.043]		
ASY										0.369**
										[1.997]
TPUT_20	0.163***	−0.036*	−19.347**	−1.373	0.156***	−2.552	0.033**	−0.629	0.878***	1.126*
	[2.619]	[-1.881]	[-2.299]	[-1.270]	[4.352]	[-1.438]	[2.530]	[-1.315]	[3.703]	[1.723]
MFB	−0.003	−0.217	−8.634***	−0.286*	0.211	0.316	0.006*	−0.140	0.005	−0.098
	[-0.459]	[-1.160]	[-2.946]	[-1.830]	[1.579]	[1.586]	[1.772]	[-0.159]	[0.079]	[-0.563]
MOB	−0.004	0.316	11.236***	0.120*	−0.368**	−0.069	−0.006	1.673*	0.196***	0.026
	[-0.970]	[1.446]	[3.216]	[1.821]	[-2.091]	[-0.324]	[-1.620]	[1.859]	[2.953]	[0.146]
Size	−0.031**	0.491***	10.773***	0.553*	−0.251	0.687**	−0.007	2.808***	−0.204***	0.409***
	[-2.182]	[2.674]	[6.521]	[1.952]	[-1.180]	[2.182]	[-1.058]	[2.671]	[-5.277]	[3.486]
Lev	0.104	0.904**	0.768	0.046	2.810**	−0.832	−0.020	1.039	−0.089	0.022
	[1.457]	[2.296]	[0.298]	[0.572]	[2.313]	[-0.610]	[-0.571]	[0.329]	[-0.778]	[0.669]
ROE	0.001	−0.002	0.196*	−0.002	−0.247	−0.052	−0.039**	5.358	0.023	−0.001***
	[0.677]	[-0.759]	[1.710]	[-0.562]	[-0.813]	[-0.504]	[-2.034]	[1.171]	[1.288]	[-2.788]
SN	−0.032*	−0.766***	−5.182**	−0.353*	−0.044	−0.453*	0.001	−4.974***	0.074	−0.760***
	[-1.949]	[-3.149]	[-2.556]	[-1.712]	[-0.215]	[-1.749]	[0.148]	[-4.834]	[1.333]	[-3.336]
Ret	1.752***	−30.840***	−190.877**	−13.088*	17.359***	−33.911***	−0.162	−140.495***	−16.730***	−30.004***
	[2.889]	[-7.019]	[-2.026]	[-1.945]	[3.571]	[-4.174]	[-1.252]	[-8.178]	[-5.441]	[-3.810]
Sigma	0.440*	6.049*	−76.463*	1.176	1.335	−0.328	0.176***	−0.747	−3.930***	8.878**
	[1.790]	[1.683]	[-1.867]	[0.327]	[0.542]	[-0.098]	[2.988]	[-0.091]	[-2.759]	[2.273]
CPI	0.637***	−5.911***	−27.315	−0.144	4.506***	−7.787***	−0.004	−48.072***	0.099	−6.773**
	[3.260]	[-3.746]	[-0.858]	[-0.058]	[2.934]	[-3.100]	[-0.079]	[-7.382]	[0.115]	[-2.572]
GDP	0.020***	−0.341***	−6.001***	−0.275**	0.292**	−0.434**	0.002	−2.284***	−0.036*	−0.188***
	[2.905]	[-4.374]	[-4.649]	[-2.049]	[2.216]	[-2.486]	[0.552]	[-4.263]	[-1.680]	[-2.592]
Constant	−2.221**	27.136***	14.053	−5.051	−19.324***	30.637***	0.165	233.838***	3.867	31.622**
	[-2.358]	[3.458]	[0.094]	[-0.404]	[-2.666]	[2.590]	[0.580]	[6.314]	[1.003]	[2.571]
	Model Criteria									
Hansen	0.158	0.133	0.0672	0.252	0.0581	0.498	0.599	0.0727	0.399	0.0723
AR(1)	0.000***	0.000***	0.000***	0.000***	0.000***	0.000***	0.042**	0.000***	0.000***	0.000***
AR(2)	0.180	0.0623	0.622	0.330	0.108	0.158	0.112	0.505	0.130	0.125
#inst	72	72	68	49	70	47	67	48	82	64
#id	4516	4516	4770	4770	4615	4617	3399	3889	4770	4770

This table presents the results of the mechanism analysis. Columns (1) and (2) evaluate the role of operating profitability as a transmission channel. Columns (3) and (4) test the mediating effect of export dependence. Columns (5) and (6) assess the impact of investor sentiment as an intermediate mechanism. Columns (7) and (8) examine the mediating effect of analyst forecast bias, while Columns (9) and (10) focus on the role of information asymmetry. T-values are clustered at the firm level and reported in brackets. *, **, and *** indicate statistical significance at the 10%, 5%, and 1% levels, respectively.

Columns (1) and (2) introduce discretionary accruals (DA) as a potential channel. Column (1) shows that TPUT_20 is positive and significant at the 5% level, indicating that trade policy uncertainty significantly raises discretionary accruals. Column (2) reports a positive and significant coefficient for DA at the 5% level. These results imply that higher discretionary accruals increase reporting opacity, enabling managers to conceal adverse information. Under rising TPU, such concealment delays market adjustment and magnifies the risk of abrupt price crashes.

Columns (3) and (4) investigate the mediating effect of export. Column (3) shows that TPUT_20 carries a significantly negative coefficient (–19.347, 1% level), indicating that rising trade policy uncertainty reduces firms’ export activity. Column (4) further reveals that Export enters positively and significantly (0.156, 5% level), suggesting that lower export intensity increases stock price crash risk. Together, these findings imply that diminished export channels weaken firms’ external demand stability, thereby amplifying their vulnerability to policy-induced crashes.

Column (5) shows that TPUT_20 is positively and significantly associated with investor sentiment (0.156, 1% level), indicating that trade policy uncertainty amplifies sentiment fluctuations. Column (6) reveals that Sentiment itself enters positively and significantly (0.276, 5% level), suggesting that heightened sentiment increases stock price crash risk. These results imply that volatile investor sentiment serves as a channel through which TPU accelerates the accumulation of mispricing, ultimately triggering crashes.

Columns (7) and (8) incorporate analyst forecast bias (FBias). Column (7) shows that TPUT_20 is positive and significant at the 5% level, indicating that trade policy uncertainty amplifies analyst forecast bias. Column (8) further reveals that FBias is positive and significant at the 5% level, suggesting that greater forecast bias increases the likelihood of stock price crashes. From an information asymmetry perspective, managerial concealment and biased analyst forecasts jointly exacerbate informational opacity, thereby reinforcing the transmission of policy uncertainty into heightened crash risk.

Finally, Columns (9) and (10) analyze the role of information asymmetry (ASY). The coefficients on TPU are 0.878 in Column (9) (1% level). ASY itself is strongly positive and significant (0.158 in Column 10, 10% level). These findings confirm that firms with higher levels of information asymmetry are more vulnerable to TPU. Under conditions of opacity and uneven information distribution, policy uncertainty triggers sharper investor reactions and accelerates crash dynamics.

The evidence confirms that TPU triggers stock price crashes through multiple mechanisms—profit manipulation, export vulnerability, sentiment fluctuations, analyst forecast bias, and information asymmetry. Together, these channels highlight how TPU undermines both firms’ fundamentals and market information environments, thereby intensifying systemic fragility.

## 5. Conclusion and policy implication

Amid rising geopolitical instability and the resurgence of protectionism, TPU has increasingly emerged as a critical external factor affecting the stability of capital markets. This study develops a novel firm-level TPU index (TPUT) using the textual content of annual reports from listed companies, and systematically evaluates its effect on stock price crash risk. The findings indicate that higher levels of TPU exposure significantly raise crash risk, a relationship that holds consistently across multiple crash risk measures (NCSKEW and DUVOL) and a battery of robustness tests. Additional heterogeneity analysis reveals that this effect is particularly pronounced among private firms and those with CEO duality, internationalized enterprises, and firms audited by non-Big Four auditors.

The analysis identifies two critical moderating mechanisms: market liberalization and digital transformation. The former mitigates risk through enhanced institutional predictability, improved disclosure quality, and more resilient financial structures. The latter enhances information-processing efficiency, strategic agility, and early warning capability, thereby reinforcing organizational adaptability to policy shocks. These findings contribute to the literature on risk response by showing how institutional and technological buffers can attenuate crash risks under external uncertainty. Mechanism analysis shows that TPU increases crash risk through multiple transmission channels: (1)earnings management via discretionary accruals, which conceals adverse fundamentals; (2) suppressed export activity, where TPU reduces firms’ access to foreign markets and damages external revenue streams, thereby heightening crash risk; (3) behavioral amplification, where investor sentiment distortions lead to herding and overreaction; (4) analyst forecast bias, which misguides expectations and reduces informational efficiency; and (5) asymmetric information, which encourages bad-news hoarding and delays timely price adjustment.

This study highlights the significant impact of trade policy uncertainty (TPU) on stock price crash risk and offers several policy recommendations to mitigate its adverse effects. At the macro level, enhancing the stability and transparency of trade policy is essential. Reducing frequent reversals in tariff schedules and strengthening credible forward guidance can narrow forecast uncertainty and stabilize market expectations. At the institutional level, deepening market liberalization and improving governance frameworks are critical. Strengthening legal protections, raising disclosure standards, and optimizing capital allocation can enhance resilience and reduce systemic fragility. Targeted reforms are also needed for vulnerable firms—such as private enterprises, internationalized firms, companies with CEO duality, and those audited by non-Big Four auditors. Policies should promote stronger governance practices, encourage the separation of CEO and board chair roles, improve disclosure discipline, and adopt higher-quality auditing standards to mitigate information opacity and bad-news hoarding. Finally, firm-level strategies are indispensable. Promoting digital transformation enhances firms’ ability to process policy signals, monitor risks proactively, and respond with agility, while export diversification and supply-chain resilience can buffer losses from adverse trade shocks.

## Supporting information

S1 File 5-data and dofile. (RAR)

## References

[1] Handley K, Limão N. Trade Policy Uncertainty. Annu Rev Econ. 2022;14(1):363–95. doi: 10.1146/annurev-economics-021622-020416

[2] Davis SJ, Liu D, Sheng XS. Economic policy uncertainty in China since 1949: The view from mainland newspapers. In: Atlanta, 2019.

[3] Baker M, Wurgler J. Investor Sentiment and the Cross‐Section of Stock Returns. The Journal of Finance. 2006;61(4):1645–80. doi: 10.1111/j.1540-6261.2006.00885.x

[4] Bianconi M, Esposito F, Sammon M. Trade policy uncertainty and stock returns. Journal of International Money and Finance. 2021;119:102492. doi: 10.1016/j.jimonfin.2021.102492

[5] Adjei F, Adjei M. Trade Policy Uncertainty, Market Return, and Expected Return Predictability. JFE. 2021;9(3):106–14. doi: 10.12691/jfe-9-3-2

[6] Zhang L, Chen W, Hu N. Economic policy uncertainty and stock liquidity: evidence from China. IJOEM. 2021;18(1):22–44. doi: 10.1108/ijoem-06-2020-0625

[7] Baker SR, Bloom N, Davis SJ. Measuring Economic Policy Uncertainty*. The Quarterly Journal of Economics. 2016;131(4):1593–636. doi: 10.1093/qje/qjw024

[8] Liu H, Yu J, Tang G, Chen J. External trade policy uncertainty, corporate risk exposure, and stock market volatility. China Economic Review. 2025;89:102331. doi: 10.1016/j.chieco.2024.102331

[9] Kim Y, Su L (Nancy), Wang Z, Wu H. The Effect of Trade Secrets Law on Stock Price Synchronicity: Evidence from the Inevitable Disclosure Doctrine. The Accounting Review. 2020;96(1):325–48. doi: 10.2308/tar-2017-0425

[10] Chiang TC, Zheng D. An empirical analysis of herd behavior in global stock markets. Journal of Banking & Finance. 2010;34(8):1911–21. doi: 10.1016/j.jbankfin.2009.12.014

[11] Chen J, Hong H, Stein JC. Forecasting crashes: trading volume, past returns, and conditional skewness in stock prices. Journal of Financial Economics. 2001;61(3):345–81. doi: 10.1016/s0304-405x(01)00066-6

[12] Luo W, Shen Z, Zhu R, Hu X. Unveiling the influence of transparency in risk communication: Shifting from information disclosure to uncertainty reduction. International Journal of Disaster Risk Reduction. 2024;104:104376. doi: 10.1016/j.ijdrr.2024.104376

[13] Fiorillo P, Meles A, Pellegrino LR, Verdoliva V. Geopolitical risk and stock price crash risk: The mitigating role of ESG performance. International Review of Financial Analysis. 2024;91:102958. doi: 10.1016/j.irfa.2023.102958

[14] Kim J-B, Li Y, Zhang L. Corporate tax avoidance and stock price crash risk: Firm-level analysis. Journal of Financial Economics. 2011;100(3):639–62. doi: 10.1016/j.jfineco.2010.07.007

[15] Jin L, Myers S. R2 around the world: New theory and new tests⋆. Journal of Financial Economics. 2006;79(2):257–92. doi: 10.1016/j.jfineco.2004.11.003

[16] Wang F, Mbanyele W, Muchenje L. Economic policy uncertainty and stock liquidity: The mitigating effect of information disclosure. Research in International Business and Finance. 2022;59:101553. doi: 10.1016/j.ribaf.2021.101553

[17] Akerlof GA. The market for “lemons”: Quality uncertainty and the market mechanism. Uncertainty in economics. Elsevier. 1978. 235–51.

[18] Hirshleifer D. Behavioral Finance. Annu Rev Financ Econ. 2015;7(1):133–59. doi: 10.1146/annurev-financial-092214-043752

[19] Glosten LR, Milgrom PR. Bid, ask and transaction prices in a specialist market with heterogeneously informed traders. Journal of Financial Economics. 1985;14(1):71–100. doi: 10.1016/0304-405x(85)90044-3

[20] O’Hara M. Presidential address: Liquidity and price discovery. The Journal of Finance. 2003;58(4):1335–54.

[21] Handley K, Limão N. Policy Uncertainty, Trade, and Welfare: Theory and Evidence for China and the United States. American Economic Review. 2017;107(9):2731–83. doi: 10.1257/aer.20141419

[22] Fan G, Wang X, Ma G. Contribution of marketization to China’s economic growth. Economic Research Journal. 2011;9(283):1997–2011.

[23] Gao B, Qin M, Xie J. Does corporate digital transformation improve capital market transparency? Evidence from China. The North American Journal of Economics and Finance. 2025;76:102363. doi: 10.1016/j.najef.2025.102363

[24] Fan G, Wang X, Zhang L, Zhu H. Report on the relative progress of marketization in various regions of China. Economic Research. 2003;3(9):r18.

[25] Li Y, Jiao W, Yang Z. Enterprise digital transformation and auditor risk decision-making. Accounting Monthly. 2023;44(19):111–9.

[26] Zhou F, Wen H. Trade policy uncertainty, development strategy, and export behavior: Evidence from listed industrial companies in China. Journal of Asian Economics. 2022;82:101528. doi: 10.1016/j.asieco.2022.101528

[27] Wang Q, Weng C. Two-way risk: Trade policy uncertainty and inflation in the United States and China. Finance Research Letters. 2024;62:105154. doi: 10.1016/j.frl.2024.105154

[28] Caldara D, Iacoviello M, Molligo P, Prestipino A, Raffo A. The economic effects of trade policy uncertainty. Journal of Monetary Economics. 2020;109:38–59. doi: 10.1016/j.jmoneco.2019.11.002

[29] Walczak N, Huremović K, Rungi A. Evaluating the EU Carbon Border Adjustment Mechanism with a Quantitative Trade Model. arXiv preprint arXiv:250623341. 2025.

[30] Gulen H, Ion M. Policy Uncertainty and Corporate Investment. Rev Financ Stud. 2015;:hhv050. doi: 10.1093/rfs/hhv050

[31] Benguria F, Choi J, Swenson DL, Xu M (Jimmy). Anxiety or pain? The impact of tariffs and uncertainty on Chinese firms in the trade war. Journal of International Economics. 2022;137:103608. doi: 10.1016/j.jinteco.2022.103608

[32] Crowley M, Meng N, Song H. Tariff scares: Trade policy uncertainty and foreign market entry by Chinese firms. Journal of International Economics. 2018;114:96–115. doi: 10.1016/j.jinteco.2018.05.003

[33] Pástor Ľ, Veronesi P. Political uncertainty and risk premia. Journal of Financial Economics. 2013;110(3):520–45. doi: 10.1016/j.jfineco.2013.08.007

[34] Du Y, Sui X, Wei W, Du J. Economic policy uncertainty and stock price crash risk. Asia-Pacific Journal of Accounting & Economics. 2021;30(3):667–89. doi: 10.1080/16081625.2021.2007407

[35] Habib A, Hasan MM, Jiang H. Stock price crash risk: review of the empirical literature. Accounting & Finance. 2017;58(S1):211–51. doi: 10.1111/acfi.12278

[36] Chung KH, Chuwonganant C. Uncertainty, market structure, and liquidity. Journal of Financial Economics. 2014;113(3):476–99. doi: 10.1016/j.jfineco.2014.05.008

[37] Cui X, Yao S, Fang Z, Wang H. Economic policy uncertainty exposure and earnings management: evidence from China. Accounting & Finance. 2020;61(3):3937–76. doi: 10.1111/acfi.12722

[38] Hutton AP, Marcus AJ, Tehranian H. Opaque financial reports, R2, and crash risk⋆. Journal of Financial Economics. 2009;94(1):67–86. doi: 10.1016/j.jfineco.2008.10.003

[39] Bloom N. The impact of uncertainty shocks. Econometrica. 2009;77(3):623–85.

[40] Bloom N. Fluctuations in Uncertainty. Journal of Economic Perspectives. 2014;28(2):153–76. doi: 10.1257/jep.28.2.153

[41] Karambakuwa RT, Ncwadi R. The Impact of United States of America-China Trade War on Market Capitalization of Emerging Economies. GTCJ. 2020;15(Issue 11/12):508–24. doi: 10.54648/gtcj2020090

[42] Meng Q, Song X, Liu C, Wu Q, Zeng H. The impact of block trades on stock price synchronicity: Evidence from China. International Review of Economics & Finance. 2020;68:239–53. doi: 10.1016/j.iref.2020.04.009

[43] Malmendier U, Pezone V, Zheng H. Managerial Duties and Managerial Biases. Management Science. 2023;69(6):3174–201. doi: 10.1287/mnsc.2022.4467

[44] Galariotis EC, Krokida SI, Spyrou SI. Bond market investor herding: Evidence from the European financial crisis. International Review of Financial Analysis. 2016;48:367–75.

[45] Chen X, Chiang TC. Empirical investigation of changes in policy uncertainty on stock returns—Evidence from China’s market. Research in International Business and Finance. 2020;53:101183. doi: 10.1016/j.ribaf.2020.101183

[46] Gleason CA, Lee CMC. Analyst Forecast Revisions and Market Price Discovery. The Accounting Review. 2003;78(1):193–225. doi: 10.2308/accr.2003.78.1.193

[47] He G, Bai L, Ren HM. Analyst coverage and future stock price crash risk. JAAR. 2019;20(1):63–77. doi: 10.1108/jaar-09-2017-0096

[48] Kim J-B, Lu LY, Yu Y. Analyst Coverage and Expected Crash Risk: Evidence from Exogenous Changes in Analyst Coverage. The Accounting Review. 2018;94(4):345–64. doi: 10.2308/accr-52280

[49] Goldstein MA, A. Kavajecz K. Eighths, sixteenths, and market depth: changes in tick size and liquidity provision on the NYSE. Journal of Financial Economics. 2000;56(1):125–49. doi: 10.1016/s0304-405x(99)00061-6

[50] He F, Lucey B, Wang Z. Trade policy uncertainty and its impact on the stock market -evidence from China-US trade conflict. Finance Research Letters. 2021;40:101753. doi: 10.1016/j.frl.2020.101753

[51] Wang H, Shen H, Tang X, Wu Z, Ma S. Trade policy uncertainty and firm risk taking. Economic Analysis and Policy. 2021;70:351–64. doi: 10.1016/j.eap.2021.03.007

[52] Ahrens J. Governance And The Implementation Of Technology Policy In Less Developed Countries. Economics of Innovation and New Technology. 2002;11(4–5):441–76. doi: 10.1080/10438590200000008

[53] Guo J, Li C, Jiao W, Wang Z. Marketisation, information transparency and the cost of equity for family firms. Finance Research Letters. 2021;38:101394. doi: 10.1016/j.frl.2019.101394

[54] Li L, Liu Q, Wang J, Hong X. Carbon Information Disclosure, Marketization, and Cost of Equity Financing. Int J Environ Res Public Health. 2019;16(1):150. doi: 10.3390/ijerph16010150 30626003 PMC6339175

[55] Vishwanath T. Toward Transparency: New Approaches and Their Application to Financial Markets. The World Bank Research Observer. 2001;16(1):41–57. doi: 10.1093/wbro/16.1.41

[56] Boehm J. The Impact of Contract Enforcement Costs on Value Chains and Aggregate Productivity. The Review of Economics and Statistics. 2022;104(1):34–50. doi: 10.1162/rest_a_00940

[57] Ryan N. Contract Enforcement and Productive Efficiency: Evidence From the Bidding and Renegotiation of Power Contracts in India. ECTA. 2020;88(2):383–424. doi: 10.3982/ecta17041

[58] Bharath ST, Pasquariello P, Wu G. Does Asymmetric Information Drive Capital Structure Decisions?. Rev Financ Stud. 2008;22(8):3211–43. doi: 10.1093/rfs/hhn076

[59] Bissoondoyal-Bheenick E, Do H, Hu X, Zhong A. Sentiment and stock market connectedness: Evidence from the U.S. – China trade war. International Review of Financial Analysis. 2022;80:102031. doi: 10.1016/j.irfa.2022.102031

[60] Arianpoor A, Mohammadbeikzade N. Stock liquidity, future investment and future investment efficiency in an emerging economy: investigating the moderator role of financial constraints. JIABR. 2023;16(4):699–721. doi: 10.1108/jiabr-07-2022-0177

[61] Molyneux P, Wang Q, Xie R, Zhao B. Bank funding constraints and stock liquidity. The European Journal of Finance. 2022;29(1):1–16. doi: 10.1080/1351847x.2022.2098046

[62] Xu B, Zhang S, Chen X. Uncertainty in financing interest rates for startups. Industrial Marketing Management. 2021;94:150–8. doi: 10.1016/j.indmarman.2020.02.026

[63] Hrytsenko LL, Zakharkina LS, Zakharkin OO, Novikov VM, Chukhno R. The impact of digital transformations on the transparency of financial-economic relations and financial security of Ukraine. 2022.

[64] Liu M, Li H, Li C, Yan Z. Digital transformation, financing constraints and enterprise performance. EJIM. 2023;28(4):1472–97. doi: 10.1108/ejim-05-2023-0349

[65] Lombardi R, Secundo G. The digital transformation of corporate reporting – a systematic literature review and avenues for future research. MEDAR. 2020;29(5):1179–208. doi: 10.1108/medar-04-2020-0870

[66] Vial G. Understanding digital transformation: A review and a research agenda. Managing digital transformation. 2021. 13–66.

[67] Ren L, Liu J, Hao Q. How digital transformation affects the cost of equity capital: the role of information disclosure quality and stock liquidity. Industrial and Corporate Change. 2024;33(5):1098–122.

[68] Wu H, Wang Y. Digital transformation and corporate risk taking: Evidence from China. Global Finance Journal. 2024;62:101012. doi: 10.1016/j.gfj.2024.101012

[69] Zhu S, Gao J, Chen K. Digital transformation and risk of share price crash: Evidence from a new digital transformation index. Finance Research Letters. 2023;58:104403. doi: 10.1016/j.frl.2023.104403

[70] Xu N, Yu S, Yi Z. Herding behavior of institutional investors and stock price crash risk. Management World. 2013;7:31–42.

[71] Wu Y, Li H, Luo R, Yu Y. How digital transformation helps enterprises achieve high-quality development? Empirical evidence from Chinese listed companies. EJIM. 2023;27(8):2753–79. doi: 10.1108/ejim-11-2022-0610

[72] Xie Q, Fang T, Rong X, Xu X. Nonlinear behavior of tail risk resonance and early warning: Insight from global energy stock markets. International Review of Financial Analysis. 2024;93:103162. doi: 10.1016/j.irfa.2024.103162

[73] Satwekar A, Miozza M, Abbattista C, Palumbo S, Rossi M. Triad of Digital Transformation: Holistic Orchestration for People, Process, and Technology. IEEE Trans Eng Manage. 2024;71:7815–31. doi: 10.1109/tem.2024.3384995

[74] Avgouleas E, Kiayias A. The Promise of Blockchain Technology for Global Securities and Derivatives Markets: The New Financial Ecosystem and the ‘Holy Grail’ of Systemic Risk Containment. Eur Bus Org Law Rev. 2019;20(1):81–110. doi: 10.1007/s40804-019-00133-3

[75] Tan S, Liu T, Wang C. The double-edged effect of bank liquidity creation efficiency on systemic risk: Evidence from China. PLoS One. 2024;19(11):e0313208. doi: 10.1371/journal.pone.0313208 39541306 PMC11563395

[76] Yang HX, Jianguo. Trade policy uncertainty and firm radical innovations—evidence from textual analysis of Chinese listed companies. International trade issues. 2024;9:87–101.

[77] Barberis N, Shleifer A, Vishny R. A model of investor sentiment. Journal of Financial Economics. 1998;49(3):307–43.

[78] Baltagi BH, Baltagi BH. Econometric analysis of panel data. Springer. 2008.

[79] Arellano M, Bover O. Another look at the instrumental variable estimation of error-components models. Journal of Econometrics. 1995;68(1):29–51. doi: 10.1016/0304-4076(94)01642-d

[80] Blundell R, Bond S. Initial conditions and moment restrictions in dynamic panel data models. Journal of Econometrics. 1998;87(1):115–43. doi: 10.1016/s0304-4076(98)00009-8

[81] Healy PM, Palepu KG. Information asymmetry, corporate disclosure, and the capital markets: A review of the empirical disclosure literature. Journal of Accounting and Economics. 2001;31(1–3):405–40. doi: 10.1016/s0165-4101(01)00018-0

[82] Yan H, Xiao W, Deng Q, Xiong S. Analysis of the Impact of U.S. Trade Policy Uncertainty on China Based on Bayesian VAR Model. Journal of Mathematics. 2022;2022(1). doi: 10.1155/2022/7124997

[83] Suwanprasert W. The international spillover effects of US trade policy uncertainty. Economics Letters. 2022;212:110286. doi: 10.1016/j.econlet.2022.110286

[84] Gao J, Zhou S. Spillover effects of US economic policy uncertainty on emerging markets: Based on transnational supply chains. Shengjie. 2025.

